# Multi-Omics Profiles of Small Intestine Organoids in Reaction to Breast Milk and Different Infant Formula Preparations

**DOI:** 10.3390/nu16172951

**Published:** 2024-09-02

**Authors:** Xianli Wang, Shangzhi Yang, Chengdong Zheng, Chenxuan Huang, Haiyang Yao, Zimo Guo, Yilun Wu, Zening Wang, Zhenyang Wu, Ruihong Ge, Wei Cheng, Yuanyuan Yan, Shilong Jiang, Jianguo Sun, Xiaoguang Li, Qinggang Xie, Hui Wang

**Affiliations:** 1 School of Public Health, Shanghai Jiao Tong University School of Medicine, Shanghai 200025, China; wangxianli@shsmu.edu.cn (X.W.); grh@shsmu.edu.cn (R.G.); mishua_cheng@163.com (W.C.); yanyuanyuan0801@outlook.com (Y.Y.); 2 School of Medicine, Shanghai Jiao Tong University, Shanghai 200025, China; ysz_aurora_soleil@sjtu.edu.cn (S.Y.); touma_kazusa@sjtu.edu.cn (C.H.); ktxaaa66@sjtu.edu.cn (H.Y.); gzm666@sjtu.edu.cn (Z.G.); 941137@sjtu.edu.cn (Y.W.); whether@sjtu.edu.cn (Z.W.); 3 Heilongjiang Firmus Dairy Co., Ltd., C-16, 10A Jiuxianqiao Rd., Chaoyang, Beijing 100015, China; zhengchengdong@feihe.com (C.Z.); jiangshilong@feihe.com (S.J.); sunjianguo@feihe.com (J.S.); 4 Institutes of Biomedical Sciences, Fudan University, 131 Dongan Road, Shanghai 200032, China; 21211510002@m.fudan.edu.cn; 5 State Key Laboratory of Systems Medicine for Cancer, Center for Single-Cell Omics, School of Public Health, Shanghai Jiao Tong University School of Medicine, Shanghai 200025, China; lixg@shsmu.edu.cn

**Keywords:** organoids, transcriptome, metabolomics, infant formulas, small intestine, growth development

## Abstract

Ensuring optimal infant nutrition is crucial for the health and development of children. Many infants aged 0–6 months are fed with infant formula rather than breast milk. Research on cancer cell lines and animal models is limited to examining the nutrition effects of formula and breast milk, as it does not comprehensively consider absorption, metabolism, and the health and social determinants of the infant and its physiology. Our study utilized small intestine organoids induced from human embryo stem cell (ESC) to compare the nutritional effects of breast milk from five donors during their postpartum lactation period of 1–6 months and three types of Stage 1 infant formulae from regular retail stores. Using transcriptomics and untargeted metabolomics approaches, we focused on the differences such as cell growth and development, cell junctions, and extracellular matrix. We also analyzed the roles of pathways including AMPK, Hippo, and Wnt, and identified key genes such as ALPI, SMAD3, TJP1, and WWTR1 for small intestine development. Through observational and in-vitro analysis, our study demonstrates ESC-derived organoids might be a promising model for exploring nutritional effects and underlying mechanisms.

## 1. Introduction

Adequate and comprehensive nutrition during infancy is crucial to ensuring the health, growth, and development of children [1,2]. Breast milk is considered the “gold standard” for infant feeding, providing a comprehensive, balanced, and easily absorbable diet of essential nutrients such as oligosaccharides, specific lipids, cytokines, growth factors, and vitamins. It promotes optimal development in infants. The benefits and necessity of breastfeeding in the early stages of an infant’s life have been widely reported [3,4,5,6,7,8,9,10].

Despite these benefits, breastfeeding may not always be feasible for various reasons. Health issues, medications that could be transmitted through breast milk, social or cultural factors, and even the infant’s medical needs for specialized formula may lead to an early weaning process or an abrupt cessation of breastfeeding [11]. We focus on infants below six months of age, and globally, only 40% of infants under six months are exclusively breastfed [12]. In this scenario, the production of infant formulae is aimed at providing comprehensive nutrition for infants, serving as a primary or unique nutritional source during the initial months of life. These products adjust their macro- and micronutrients to mimic breast milk in either composition or performance, while the heterogeneity in breast milk across different donors and feeding stages brought challenges [13,14,15,16,17]. Some products also attempt to alter their nutrition profile to fill the nutrition needs of specific infant populations (e.g., those with allergies to certain milk ingredients, preterm infants, and metabolic abnormalities).

Furthermore, the gastrointestinal tract of infants aged 0–6 months is still in the developmental stage, with immature development, low levels of digestive juices, enzyme activity, and gastrointestinal motility [18]. As a result, issues related to low digestive function due to insufficient absorption of proteins, lipids, and lactose or digestive problems caused by imbalances in the intestinal microbiota often occur during this period. However, infant formula differs from breast milk in terms of protein composition and content, microscopic structure of fat globules, lipid components, and structure. Additionally, due to cost considerations, infant formula often lacks human milk oligosaccharides (HMOs) found in breast milk, and substitutes such as fructooligosaccharides or galactooligosaccharides are used. The levels of immunoglobulins, antimicrobial peptides, growth factors, and other components in infant formula are also often relatively lower compared to breast milk [19,20,21]. Additionally, substances such as DHA (Docosahexaenoic acid) and ARA (arachidonic acid) have been widely added to infant formula in recent years. An early study from 1992 on infant brain autopsies showed that the average FA% (percent of fatty acids) of DHA in the cerebral cortex phospholipids of breastfed infants (9.7%) was approximately 25% higher compared to formula-fed infants (7.6%) [22,23,24]. To sum up, there is still a big gap between formula and breast milk, and scientists in the field have never stopped trying to reduce the gap.

In the late 20th century and early 21st century, classical cell lines and animal model systems achieved success in many areas of biological and medical research, including studies on the small intestine. Examples include CACO-2 (human colorectal adenocarcinoma cell line, simulating intestine epithelial cells), LS174T (human adenocarcinoma-derived, simulating intestine goblet cells), and mouse models, which were widely utilized. However, differences exist between animal models and human early developmental physiology; for instance, the timing of the differentiation of Paneth cells in the small intestine varies between humans and mice [25]. Moreover, model animals like mice and the associated facilities can be expensive. The use of cancer cell lines derived from cancer tissues for the study of normal tissues also has its own limitations. For instance, CACO-2 cells lack the expression of certain specific transport proteins and metabolic enzymes [26,27,28,29], and the tightness of cell connections in CACO-2 cells is significantly stronger compared to the in vivo small intestine [30]. This indicates that cancer cell lines may not fully represent the physiology of the small intestine. Moreover, animal models and cancer cell lines have their limitations, as limitations in translating clinical data from animal models and cancer cell lines may cause failure of ∼90% of all potential drug therapies in human clinical trials [31,32]. And organoids, as an emerging technology, have great potential. Organoids recapitulate the in vivo tissue structure, heterogeneity of multiple cell types, and interactions observed in vivo. Therefore, they may more accurately simulate human tissue functionality and physiology compared to animal models or 2D models [33,34].

For the study of the small intestine in early infant life, organoids induced from human embryonic stem cells (ESCs) have homogeneity with normal infant intestinal development and similarity in developmental stages. Organoids derived from human pluripotent stem cells are suitable for simulating the physiology or diseases of the early infant, as they resemble developing fetal tissues [35,36,37,38]. Therefore, we hypothesize that small intestine organoids are an ideal material for studying nutrient absorption in the infant intestinal tract during early life.

However, there is a lack of research utilizing small intestine organoid models in the dairy research field. In this study, intestine organoids induced from human ESCs were used to compare the differences and common characters in absorption and metabolism between breast milk samples and formula milks. The three formulas (Stage 1 infant formulas) were sampled from a segment of milk powder of the same brand (Firmus, China), which constituted the same main components and different special additions (see Table S1 for ingredients). Non-targeted metabolomics and transcriptomics methods were integrated to investigate the nutritional effects of breast milk and formula.

## 2. Materials and Methods

### 2.1. Breast Milk Donors

Breast milk donors were screened as described by Spitzer et al. [39], and breast milk samples were collected from a total of five eligible donors. These donors (Chinese, Han nationality) were between 25 and 31 years of age, with a mean age of 28 years, and samples were collected during their postpartum lactation period of 1 month to 6 months. The breast milk donors produced more milk than their infants needed. They were all in good health, with normal milk production, no history of mastitis, breast trauma, or abscesses, no history of medication use during lactation, and no adverse lifestyle habits such as smoking or alcohol consumption. All breast milk donors were fully informed of the purpose and nature of the study and signed an informed consent form prior to sample collection and analysis. This research was affiliated with an observational study (ClinicalTrials.gov identifier: NCT05133466) sponsored by Heilongjiang Firmus Dairy Co., Ltd. (Qiqihaer, China) and was ethically approved by the Ethics Committee of Peking University (approval number IRB00001052-21091).

### 2.2. Breast Milk Collection

Breast milk sample collection and storage procedures followed general recommendations for the use of breast milk handling in the home of healthy infants [40]. Meanwhile, in order to simulate the common behavioral patterns of breastfeeding, all collected breast milk samples were a mixture of foremilk and hindmilk [40]. Sampling (100∼160 mL) was performed approximately one to two hours after the donor’s morning breastfeeding. During the sampling, the entire breast milk of the non-feeding side of the breast was aspirated using an electric or mechanical breast pump, depending on the preference and habits of each donor. The collected breast milk samples were stored in sterile polyethylene HM bags and immediately frozen on dry ice (−78 °C) and then transported to the laboratory for storage in a −80 °C freezer for subsequent analysis.

### 2.3. Infant Formulas

The stage-1 infant formulas used for this study were purchased in a local supermarket in Shanghai, China. In this study, stage-1 means this kind of infant formulas were designed for infants of 0–6 months, as the concept of “staging” on infant formulas has been previously proposed by early studies [15,19]. Three types of stage-1 infant formulas were used, which are three sub-brands from one brand (Firmus, China), thus having the same main ingredients and different extra additive ingredients (detailed information see Table S1). Before reconstitution, infant formulae were stored at 4 °C. The ratio of dissolved dry weight to water of reconstitution follows the guidelines provided in the product manual.

### 2.4. Induced Differentiation of Small Intestine Organoids

We used the human embryonic stem cell line H1, which was obtained from WiCell (https://www.wicell.org/?option=com_oscommerce&Itemid=192, accessed on 15 July 2020). Human intestinal organoids were generated and maintained as previously described [41,42,43]. Human ESCs and induced pluripotent stem cells were seeded at a density of 100,000 cells per well on a 24-well Nunclon surface plate (Nunc) coated with Matrigel (BD Bioscience, Franklin Lakes, NJ, USA). The cells were treated with 100 ng/mL Activin A in RPMI 1640 (Invitrogen, Gibco, Thermo Fisher Scientific, Waltham, MA, USA) for three consecutive days to form definitive endoderm, with a gradual increase in tetracycline-free fetal bovine serum (0%, 0.2%, and 2% concentrations on the first, second, and third days, respectively). During the definitive endoderm induction process, a control without Activin A was included to monitor endoderm differentiation efficiency. Subsequently, the cells were incubated for four days in DMEM-F12 containing 2% tetracycline-free fetal bovine serum, 400 ng/mL FGF4, and 3 mM CHIR99021 (Stemgent, Selleck, Houston, TX, USA) for hindgut differentiation. The mid-hindgut spheroids were then collected, embedded in 50 μL Matrigel (BD Bioscience, Minneapolis, MN, USA), and maintained under conditions containing 500 ng/mL R-Spondin1 (R&D Systems, Franklin Lakes, NJ, USA), 100 ng/mL Noggin (R&D Systems, Minneapolis, MN, USA), and 50 ng/mL EGF (R&D Systems, Minneapolis, MN, USA). After Matrigel solidification, the medium (Advanced DMEM/F12 supplemented with L-glutamine, 10 μM Hepes, N2 supplement (R&D Systems, Minneapolis, MN, USA), B27 supplement (Invitrogen, Gibco, Thermo Fisher Scientific, Waltham, MA, USA), and Pen/Strep with growth factors) was added, and the medium was changed every four days.

### 2.5. In Vitro Digestion Simulation of Milk

A 25 μL solution of 0.3 M CaCl_2_ was first prepared, then added to 975 μL of water. A 0.5 mL sample of 1500 U/mL salivary α-amylase and simulate salivary fluid (SSF) were added by magnetic stirring at 37 °C for 25 min. 0.2 g NaCl and 1.3 mL of 1 mol/L HCl were dissolved in 80 mL of water, then adjusted the pH to 5.5 with 0.1 mol/L HCl, making up the solution to 100 mL. Then gastric lipase (1.3 mg/mL) and pepsin (1.5 mg/mL) were added while stirring magnetically at 37 °C for 25 min; thus, simulated gastric fluid (SGF) was obtained. A solution of 164 mmol/L NaCl, 10 mmol/L KCl, and 119 mmol/L CaCl_2_ was prepared, and its pH was adjusted to 6.5 with 0.2 mol/L NaOH, then adding bile salt (0.5 mg/mL) and pancreatin (1 mg/mL) while stirring magnetically at 37 °C for 25 min to simulate intestinal fluid (SIF). A 5 mL sample (breast milk or formula solution, standardized by dry weight to 130 g/L) was taken and placed in a 37 °C water bath for 10 min. A 5 mL SSF was added, and the sample was stirred at 37 °C for 1 min. A 10 mL SGF was then added and titrated with 0.1 mol/L NaOH or HCl to maintain the system pH at 5.5. The samples were digested for 30 min. Then the pH of the simulated gastric fluid digestion solution was adjusted to 6.5 with 0.2 mol/L NaOH. A 10 mL SIF was added, and samples were digested for 2 h. During this period, samples were titrated with 0.1 mol/L NaOH to maintain the pH at 6.5.

### 2.6. Establishment of the Absorption Metabolism Model

After 10 days of dormant culture in the Matrigel, the small intestine organoids were removed from the Matrigel, digested with 0.25% trypsin at 37 °C for 2 min, and cells were collected by centrifugation at 300 g for 5 min. Single-cell suspensions derived from organoids were plated on Polyester (PET) membrane Transwell inserts with a 0.4-mm pore size (Corning Life Sciences, Corning, NY, USA) at a concentration of 10 cells/mL. Complete culture medium for small intestine organoids was added. The media was changed every other day. When the culture reached confluence, based on transepithelial electrical resistance (TEER) monitoring and direct observation under a microscope (approximately 10 days), the medium was changed to DMEM/F12 with added growth factors, B27-free medium was used, and cells were subjected to 12 h of starvation treatment to remove previous growth factors’ effects on promotion of proliferation and differentiation. After that, the sample (breast milk or formula) digestion solution was added (1 mL digestion solution to 1 mL starvation medium each), and after 12 h, the small intestine organoid cells were collected for subsequent analyses. Cells treated with starvation served as the negative control. Collected samples were stored in a −80 °C freezer.

### 2.7. RNA Sequencing

Total RNA was extracted from the samples using Trizol, and the quality of the extracted total RNA samples was assessed using the Agilent RNA Nano assay kit (Agilent, Santa Clara, CA, USA). Following the manufacturer’s instructions, the MGIEasy RNA Library Prep kit was utilized for library preparation, using a total of 200 ng of RNA per sample. In summary, after mRNA enrichment, the samples were incubated with a fragment buffer at 94 °C for 8 min to obtain target insert fragments of approximately 150 bp. Subsequently, the products were reverse transcribed into cDNA. After repair and A-tailing, the double-stranded cDNA products were ligated with adapters, and PCR amplification was carried out for 14 cycles (95 °C for 30 s, 56 °C for 30 s, 72 °C for 60 s; 14 cycles). After purification of the PCR products using DNA Clean Beads, the quality of the purified PCR products was assessed using the Agilent DNA 1000 assay kit. The PCR products with a fragment size of approximately 230 bp were pooled, circularized, and enzymatically cut to obtain the resulting library. Subsequently, analysis was performed on the Illumina NextSeq 500 using a single-end 50 bp module. Raw data (raw reads) in Fastq format underwent initial quality control using FastQC, followed by alignment of reads using Hisat2 (version 2.2.1) and quantification using StringTie to obtain raw counts.

### 2.8. Untargeted Metabolite Sequencing

The analysis was conducted using a UHPLC-Q-TOF MS system. After slow thawing at 4 °C, an appropriate amount of sample (50 μL cell pellet placed in a 1.5 mL centrifuge tube) was added to a pre-cooled solution of methanol/acetonitrile/water (2:2:1, *v*/*v*), vortex-mixed, sonicated at 4 °C for 30 min, allowed to stand at −20 °C for 10 min, and centrifuged at 14,000× *g* at 4 °C for 20 min. The supernatant was vacuum-dried, and for mass spectrometry analysis, 100 μL of acetonitrile-water solution (acetonitrile: water = 1:1, *v*/*v*) was added for reconstitution, vortex-mixed, and centrifuged at 14,000× *g* at 4 °C for 15 min. The supernatant was then subjected to injection for analysis.

Chromatographic conditions: The samples were separated using an Agilent 1290 Infinity LC ultra-high-performance liquid chromatography system (UHPLC) with an HILIC column; column temperature, 25 °C; flow rate, 0.5 mL/min; injection volume, 2 μL; composition of mobile phases: A—water + 25 mM ammonium acetate + 25 mM ammonia solution, B—acetonitrile. The gradient elution program was as follows: 0–0.5 min, 95% B; 0.5–7 min, linear decrease of B from 95% to 65%; 7–8 min, linear decrease of B from 65% to 40%; 8–9 min, maintenance of B at 40%; 9–9.1 min, linear increase of B from 40% to 95%; 9.1–12 min, maintenance of B at 95% (see Table S2). The samples were kept in an automatic sampler at 4 °C throughout the entire analysis process. To avoid the influence of fluctuations in instrument detection signals, samples were continuously analyzed in a random order. QC samples were inserted into the sample queue to monitor and evaluate the stability of the system and the reliability of the experimental data.

Q-TOF MS conditions: An AB Triple TOF 6600 mass spectrometer was used for the acquisition of primary and secondary spectra. After separation using the Agilent 1290 Infinity LC UHPLC system, the Triple TOF 6600 mass spectrometer (AB SCIEX, Toronto, Canada) was employed for mass spectrometric analysis in positive and negative ion modes using electrospray ionization (ESI). ESI source settings were as follows: Gas1 (nebulizer gas) at 60, Gas2 (heater gas) at 60, curtain gas (CUR) at 30 psi, ion source temperature at 600 °C, and spray voltage (ISVF) at ±5500 V (positive and negative modes). The detection range for primary mass-to-charge ratio was 60–1000 Da, and for secondary daughter ions was 25–1000 Da. The accumulation time for primary mass spectrometry was 0.20 s/spectra, and for secondary mass spectrometry, it was 0.05 s/spectra. Secondary mass spectrometry was performed in data-dependent acquisition mode (IDA) using peak intensity value filtering. The declustering voltage (DP) was set at ±60 V (positive and negative modes), collision energy at 35 ± 15 eV. The IDA settings included a dynamic exclusion range of 4 Da, and 10 fragment spectra were collected per scan.

### 2.9. Data Analysis of Transcriptome

Gene normalization, batch effect removal, and differential analysis were performed using DESeq2. The quantified expression data was obtained using the rlog algorithm provided by DESeq2 and was subsequently utilized for additional analyses such as principal component analysis (PCA) and others [44]. Gene overrepresentation analysis and GSEA (gene set enrichment analysis) and related normalized enrichment score (NES, representing the degree of enrichment) calculations were conducted by the R package clusterprofiler [45]. GSVA was conducted by the GSVA [46] R package. GO enrichment in the form of a function relation network was performed using a Cytoscape plug-in clueGO [47,48]. Pathway enrichment metrics z-score formula and dendrogram method were offered in GeneTonic [49]. The visualization of GSEA (Gene Set Enrichment Analysis) results is achieved using the R package GseaVis (version 0.0.5). The protein–protein association network was generated by STRING (STRING: functional protein association networks (https://cn.string-db.org, accessed on 15 December 2023) [50], and the genetic regulating network was generated by IPA (ingenuine pathway analysis, QIAGEN, Fall Release, 2023) software [51].

### 2.10. Data Analysis of Untargeted Metabolomics

The raw MS data were converted to MzXML files using ProteoWizard MSConvert before importing into freely available XCMS (version 4.2.3) software. For peak picking, the following parameters were used: centWave *m*/*z* = 10 ppm, peakwidth = c (10, 60), prefilter = c (10, 100). For peak grouping, bw = 5, mzwid = 0.025, and minfrac = 0.5 were used. CAMERA (collection of algorithms of metabolite profile annotation) was sued for annotation of isotopes and adducts. In the extracted ion features, only the variables having more than 50% of the nonzero measurement values in at least one group were kept. Compound identification of metabolites was performed by comparing the accuracy *m*/*z* value (<10 ppm) and MS/MS spectra with an in-house database established with available authentic standards.

Metabolomic data were standardized using the median fold change. The OPLS-DA (Orthogonal Partial Least Squares Discriminant Analysis) and the acquisition of model variable (metabolite) VIP (Variable Importance in Projection) values, along with the data for generating S-plots, were implemented using the R package ropls [52]. Limma [53] was also employed for the identification of differentially expressed metabolites. Through KEGG (kyoto encyclopedia of genes and genomes) API (https://www.kegg.jp/kegg/rest/keggapi.html, accessed on 15 December 2023) to get Module: 01100 Metabolic Pathways’ all metabolites, which were all the compounds engaged in metabolic processes according to the KEGG database, the further selection of annotated compounds was conducted. Metabolites with *p*-values less than 0.05 from the limma and VIP values greater than 1 are considered differentially expressed metabolites. PLSDA was conducted by R package mixOmics [54]. Metabolite pathway analysis was conducted using the pathway analysis module in MetaboAnalyst [55].

### 2.11. Data Processing and Statistical Analysis

The heatmap was generated using the R package pheatmap; Venngraph was jenerated by jVenn [56]. Other graphs were generated by R package ggplot2. Conducted with Prism Version 10.1.0 (GraphPad Software Inc., San Diego, CA, USA), the Shapiro–Wilk test was used to verify the normality of the distribution of continuous variables, the Levene test was used for testing the homogeneity of variances, and one-way welch–ANOVA analysis was conducted. The correlation calculations were performed using the Python packages NumPy (version 1.26.0) and scipy.stats (scipy version 1.10.1), while the visualization was achieved using the R package corrplot. Spearman rank correlations were used for continuous variables. A two-sided *p*-value (adjusted) of 0.05 was considered the threshold of significance. *p* values are adjusted by Benjamini–Hochberg method. For RT-qPCR data, results are expressed as means ± SEM (standard error of the mean) with welch-ANOVA and multiple comparison tests using Prism version 10.1.0 as described above.

### 2.12. RT-qPCR

According to the manufacturer’s instructions, RNA was isolated from cells using the MiniBEST Universal RNA Extraction Kit (TaKaRa Biomedical Technology, Beijing, China). The extracted RNA was reverse transcribed into cDNA using the FastKing One Step RT-qPCR kit (FP313, SYBR Green, Tiangen Biotech, Beijing, China). The qPCR reactions were conducted using the ABI PRISM 7300 system (Applied Biosystems, Foster City, CA, USA). Each PCR cycle included an initial denaturation step at 95 °C for 5 min (1 cycle), followed by amplification cycles of denaturation at 95 °C for 30 s, annealing at 60 °C for 30 s, and extension at 72 °C for 40 s (35 cycles), with fluorescence acquisition. Comparative analysis (2^−ΔΔCt^) was performed using ACTIN as internal reference genes. The forward and reverse qPCR primers used in the study are listed in (Table S3).

## 3. Results

### 3.1. Transcriptome Profiles of Organoids Feeding by Breast Milk and Different Infant Formulas

**Figure 1:**
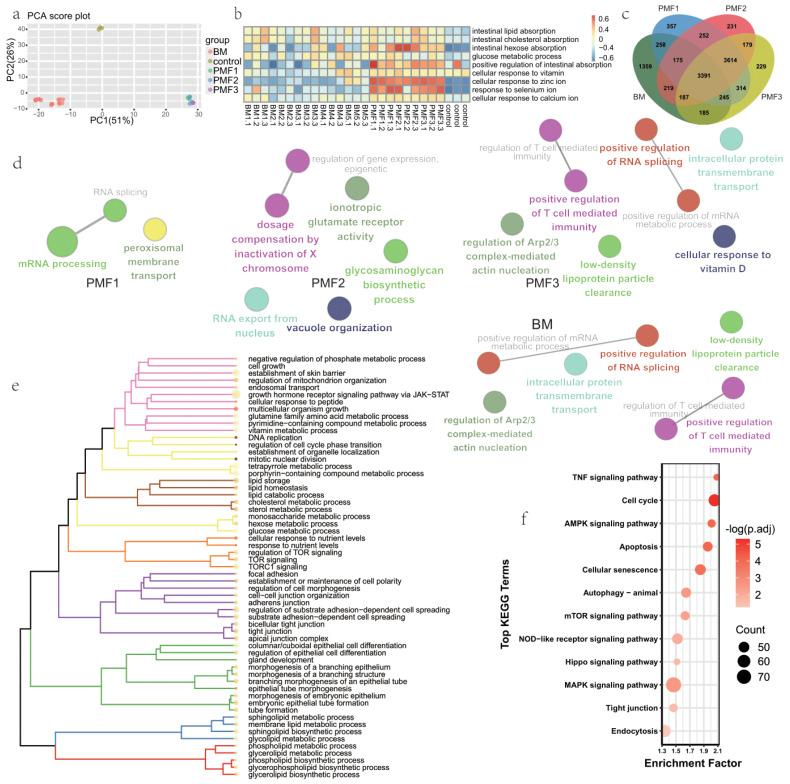
Transcriptome profiles of intestine organoids feeding by different infant formulae and breast milk. (**a**) PCA of samples in groups BM, PMF1, PMF2, PMF3, and control. PC1 and PC2 Scores of different samples are visualized, and the variance contributed by its corresponding component is presented. (**b**) GSVA analysis of each sample for GO terms associated with nutrition absorption in small intestine. (**c**) Venn graph of different groups’ DEG. (**d**,**e**) Gene over-representation analysis of GO of (**d**) unique DEG of infant formulae group and breast milk group presented in functionally grouped network with terms as nodes linked based on their kappa score level (≥0.3) using a Cytoscape plug-in clueGO, and (**e**) shared DEG of all infant formulae and breast milk presented in dendrogram using methods adopted by GeneTonic. (**f**) Pathway enrichment analysis of KEGG for shared DEG of all infant formulae and breastmilk. Top 12 enriched significant pathways (*p* value < 0.05) ordered by count were presented.

To investigate the absorption patterns and subsequent effects of the intestine on breast milk and various infant formulas, transcriptome analysis was conducted. After normalization of gene expression, gene expression of each sample was visualized (Supplementary Figure S1a,b). After performing principal component analysis (PCA) on the normalized gene expression levels obtained from all samples, scores of first and second principal components were visualized, and the similarities and differences among various samples were intuitively observed (Figure 1a). The PCA revealed significant differences between the control group, breast milk (BM) group, and infant formula (PMF) group, while differences within the infant formula group were relatively less pronounced. Gene Set Variation Analysis (GSVA) was conducted on the expression matrix of each sample, focusing on GO (gene ontology) terms related to nutrient absorption (Figure 1b). The results indicated that, in terms of lipid absorption, formula-fed infants exhibited similarities with breastfed infants. However, in the absorption and metabolism of sugars, vitamins, and specific mineral ions such as zinc, selenium, and calcium, formula-fed infants might have better performance than breast-milk-fed infants. Subsequently, differential analysis was conducted for each treated group compared to the control group. Compared to the control group, BM, PMF1, PMF2, and PMF3 identified 6019, 8606, 8248, and 8344 differential genes, respectively (volcano plots presented in Supplementary Figure S1c). The Venn diagram of DEG, which satisfies |log_2_FoldChange| > 1 and p.adjust < 0.05, revealed that among the differential genes, 3391 were shared, while BM, PMF1, PMF2, and PMF3 had 1359, 357, 231, and 229 unique differential genes, respectively (Figure 1c).

In order to understand the biological processes in which the above genes are involved, pathway enrichment analysis was applied to the shared and unique genes, respectively. The major GO terms enriched of PMF1, 2, 3, and BM’ unique differential expressed genes (DEG) were shown (Figure 1d). For unique PMF1-DEG, terms of mRNA processing and peroxisomal membrane transport are revealed. Unique PMF2-DEG’s effect concentrated on events like ionotropic receptor activity, glycosaminoglycan biosynthetic process, RNA export from the nucleus, and vacuole organization. Unique DEG of PMF3 primarily participated in processes such as RNA splicing regulation, positive regulation of T cell-mediated immunity, cellular response to vitamin D, intracellular protein transmembrane transport, low-density lipoprotein particle clearance, and regulation of Arp2/3 complex-mediated actin nucleation, which drives processes like endocytosis and cell migration (lamellipodial protrusion) [57]. We then focused on the enriched GO terms of shared 3391 genes (Figure 1e). Terms are enriched in nutrition and metabolism processes like TOR signaling, lipid metabolism, cell division and differentiation process, cell adhesion and junction, and tissue/organ development, indicating infant formulae and breastmilk’ shared function in promoting and supporting infant growth. Terms of KEGG pathways were also enriched, and these terms suggested the pathways contributing to the common effects of infant formulae and breastmilk feeding (Figure 1f). Pathways including AMPK, mTOR, TNF, and NOD-like signaling were significantly enriched, which essentially cover the various aspects of nutrition absorption and its related effects on the infant small intestine.

### 3.2. Metabolite Profiles of Breast Milk and Different Infant Formulas Treated Cells

**Figure 2:**
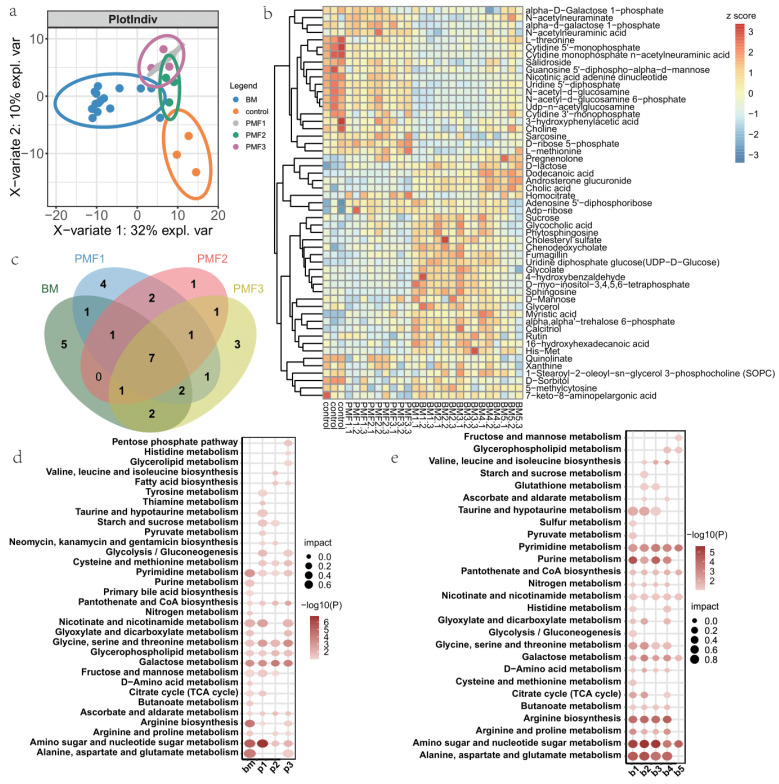
Metabolite profiles of breast milk and different infant formulae. (**a**) Inter-group PLSDA (Partial Least Square Discriminant Analysis). Variation contribution of each component was presented. (**b**) A hierarchical clustered heatmap of different metabolites identified by ANOVA analysis. (**c**) Venn plot of enriched pathways of differential metabolites of BM, PMF1, PMF2, and PMF3. (**d**) KEGG pathways enriched from differential metabolites of BM, PMF1, PMF2, and PMF3. Pathways satisfying *p* value < 0.1 were presented. (**e**) KEGG pathways enriched from differential metabolites of different breast milk groups. Pathways satisfying *p* value < 0.1 were presented.

We obtained annotated compounds involved in metabolic processes and then conducted partial least squares discriminant analysis (PLSDA). The score plot was showing inter-group differences, and all treated groups were clearly separated from the control group (Figure 2a). Through ANOVA analysis, we identified metabolites that were differentially enriched in breast milk and formula (Figure 2b). To further explore the differences among various formula types, we conducted orthogonal partial least squares discriminant analysis (OPLS-DA), visualizing scores of each sample on the predictive and orthogonal components given by the model, revealing distinct separation in the OPLS-DA plots for different formula types. No overlap was observed in both positive and negative ion modes (Supplementary Figure S2a–c), indicating differences in the metabolic profiles of various formula types. Additionally, the S-plot following OPLS-DA showcased metabolites that played a crucial role in the classification model (Supplementary Figure S2d). For the comparison between PMF1 and PMF2, the model identified dipeptides Pro-Trp, hypoxanthine, DL-cysteine, lactose, cis, and cis-muconic acid. Xanthine is involved in nucleotide metabolism, and studies have suggested that xanthine supplements can improve intestinal barrier function and wound healing [58]. In the comparison between PMF1 and PMF3, the model identified stearic acid and palmitic acid. In the comparison between PMF2 and PMF3, the model identified DL-cysteine, cis, cis-muconic acid, pro-trp, and salicylaldehyde. Through Spearman correlation analysis, metabolites that were mutually correlated in the three pairwise comparisons could be determined. The Spearman correlation of inter-infant-formulae comparison was visualized (Supplementary Figure S3a–c). For example, taurin, nicotinic acid adenine dinucleotide, and 2′−deoxyuridine 5′−monophosphate are correlated differential metabolites from comparison of PMF1 and PMF2. Other correlated differential metabolites include: malate, N−acetyl−l−aspartic acid, and D-glutamine (PMF1 vs. PMF3); N−acetyl−l−aspartic acid, D−glutamine, and Creatine (PMF2 vs. PMF3). Pathway analysis for the differential metabolites in the three pairwise comparisons is presented (Table S4). Compared to PMF1, PMF2 enriched taurine and hypotaurin metabolism pathway (*p* = 0.0391). Compared to PMF2, PMF3 enriched lactose synthesis (*p* = 0.0071) and galactose metabolism (*p* = 0.0471). Compared to PMF1, PMF3 enriched malate-aspartate shuttle (*p* = 0.0203), glucose-alanine cycle (*p* = 0.0337), citric acid cycle (*p* = 0.0339), glucogenesis (*p* = 0.0368), and glycine and serine metabolism (*p* = 0.0414).

We then conducted functional analysis of pathways enriched from differential metabolites of each group. Gathering all differential metabolites of breast milk as one group, 19, 19, 14, 18 KEGG pathways were enriched from metabolites of BM, PMF1, PMF2, and PMF3, respectively. A Venn plot of these pathways was presented, and 7 shared pathways and the unique pathways for each group: 5 (BM), 4 (PMF1), 1 (PMF2), and 3 (PMF3) were identified (Figure 2c). All four groups were enriched in glycerophospholipid metabolism as well as ascorbate and aldarate metabolism by KEGG analysis (Figure 2d). PMF1 features pathways including thiamine metabolism and niacin and niacinamide metabolism. PMF2 features Valine, leucine, and isoleucine biosynthesis and fatty acid biosynthesis. PMF3 features Arginine biosynthesis and Alanine, aspartate, and glutamate metabolism. Inspired by the previous PLS-DA plot, we inferred that there is relatively strong heterogeneity among different breast milk groups. The heatmap of metabolites for different breast milks also indicated the heterogeneity between breast milks (Supplementary Figure S3d). Therefore, we conducted KEGG pathway enrichment analysis for the differential metabolites in different breast milk groups (Figure 2e). For example, BM1, BM3, and BM5 were not enriched in ascorbate and aldarate metabolism, BM2 was not enriched in histidine metabolism, and BM4 was not enriched in taurine and hypotaurin metabolism.

### 3.3. Pro-Development Effects of Breast Milk and Different Infant Formulae on Intestine Organoid

**Figure 3:**
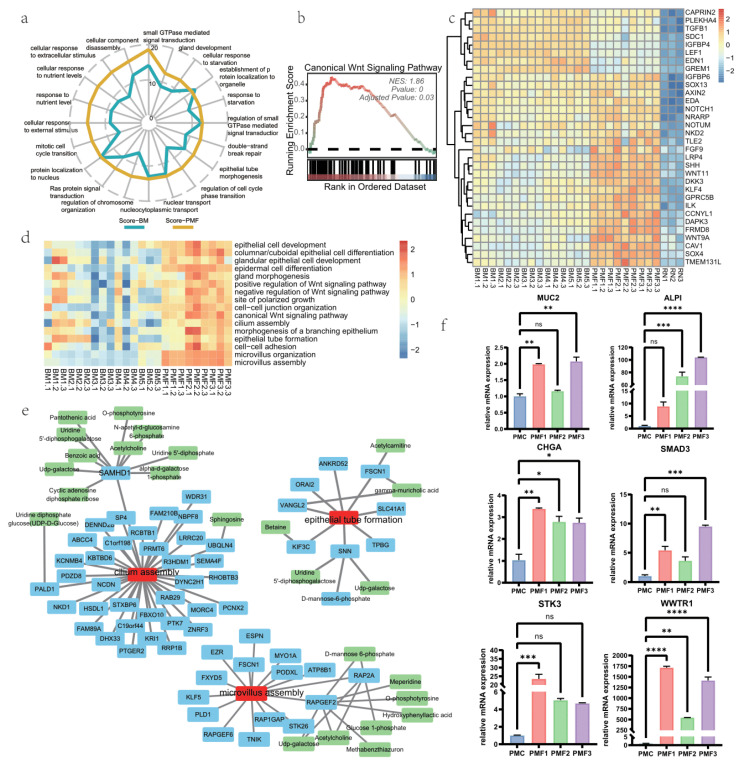
Pro-development effects of breast milk and different infant formulae on intestine organoids. (**a**) Radar plot of GO-enriched pathways’ z-score of breast milk group and PMF group. Methods are from GeneTonic, an R package for RNA-seq data. Z-score implies the intensity and direction of pathway enrichment. PMF means powder milk (infant formulae), gathering PMF1, PMF2, and PMF3 as one group. (**b**) GSEA results of GO: Canonical Wnt signaling pathway from shared DEG of BM, PMF1, PMF2, and PMF3. NES means normalized enrichment score. (**c**) Heatmap of core enrichment genes of GSEA: canonical Wnt signaling pathway. (**d**) Heatmap of GSVA score of selected pathways associated with growth and development of intestine for each sample. (**e**) Cilium assembly and epithelial tube formation’ highly correlated genes (GSVA score-gene, spearman correlation > 0.8) and the metabolites highly correlated to them (gene-metabolite, spearman correlation > 0.8). (**f**) mRNA expression level of ‘key genes’ measured by RT-qPCR technique (2^−ΔΔCt^ method). All data are presented as mean ± SEM. * *p* < 0.05; ** *p* < 0.01; *** *p* < 0.001; **** *p* < 0.0001; ns, no significance, *p* ≥ 0.05.

The early stages of human development are critically influenced by nutritional factors, which can have lasting impacts on an individual’s health trajectory. We conducted a more in-depth exploration of the effects of formula and breast milk on growth and development. Initially, we treated all formula samples as a group named PMF, performed differential analysis and pathway enrichment, and then compared them with the BM group. We found that, based on the z-score provided by GeneTonic in relevant pathways, the overall performance of formula was better than breast milk (Figure 3a). Next, we performed GSEA-GO pathway enrichment on the common DEGs obtained from breast milk and different formula groups, revealing a significant analysis of the canonical Wnt signaling pathway (Figure 3b,c). This indicates a shared promoting effect on growth between formula and breast milk.

For a more detailed comparison between formula and breast milk, as well as among different formula groups, we used the sample-wise pathway enrichment method GSVA. The GSVA score heatmap showed the degree of enrichment for each sample in a specific pathway (Figure 3d). Overall, the performance of formula was superior to the breastmilk group. Consistent with the discussion on the metabolome, breast milk exhibited a certain degree of heterogeneity. For the internal comparison of formulas, PMF2 and PMF3 were relatively superior to PMF1. We focused on cilium assembly and epithelial tube formation pathways and found that PMF2 and PMF3 had more upregulated genes compared to PMF1 (Supplementary Figure S4a,c).

Furthermore, we explored the relationship between pathways, genes, and metabolites (Figure 3e). Notably, the SAMHD1 gene associated with cilium assembly and metabolites showed a strong statistical correlation (spearman rho > 0.8, p.adj < 0.05). These metabolites include pantothenic acids, acetylcholine, and UDP-galactose (Uridine diphosphate galactose). Furthermore, the RAP2A and RAPGEF2 genes associated with microvillus assembly also exhibited significant correlations with numerous metabolites such as d-mannose 6-phosphate, acetylcholine, and glucose 1-phosphate. Subsequently, we validated the expression of key genes in formula groups using RT-qPCR (Figure 3f). Expression of MUC2, ALPI, CHGA, SMAD3, STK3, and WWTR1 was validated, and comparisons were conducted between formulas and the control group. We showed that these genes’ mean expression values of formulas were generally greater than control group, as only PMF1-ALPI, PMF2-MUC2, PMF2-SMAD3, PMF2-STK3, PMF3-STK3′ comparison was not significant. What is more, the RT-qPCR detection for various BM groups revealed significant heterogeneity among different breast milk-fed groups (Supplementary Figure S4b). Some breast milk-fed groups did not exhibit a pronounced promoting effect on the expression of developmental genes, and in some cases, even showed inhibitory effects. Exploring the regulatory mechanisms causing differences in the pro-development effects between groups is interesting but challenging. Predictions from IPA regarding the distinct regulatory networks in growth and development for each group provide us insights (see Supplementary Figure S5a–d).

### 3.4. Effects on Cell Junction of Different Infant Formulae and Breast Milk

**Figure 4:**
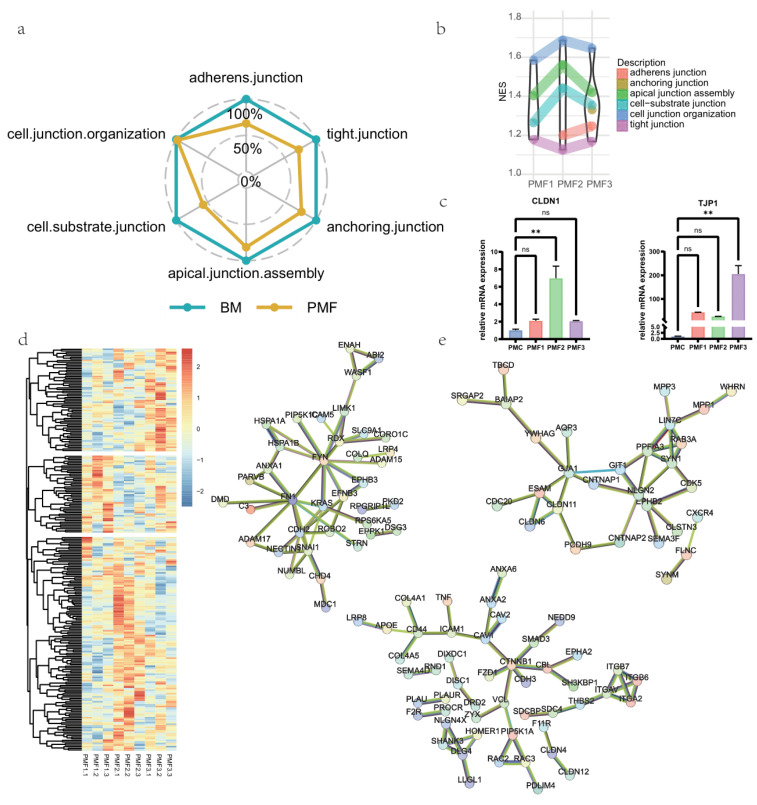
Breast milk and different infant formulae effects on cell junction assembly and regulation. (**a**) NES value of GSEA enrichment for breast milk and infant formulae. Here, treat infant formulae as one group: PMF. (**b**) Inter-PMF comparison of cell junction-related GO biological processes enriched by GSEA. NES of each GO term for each infant formula group was visualized. (**c**) mRNA level of claudin-1 (CLDN1) and ZO-1 (TJP1) quantified by RT-qPCR (2^−ΔΔCt^ method). (**d**) Hierarchical clustered heatmap of expression of core enrichment genes in tight junction pathway. Genes were clustered in three modules, representing certain infant formula groups’ relatively higher expressed genes. (**e**) Protein–protein interactions, respectively, from each clustered module’s genes. Left-Up: from module representing PMF1. Right-Up: from module representing PMF3. Middle-Down: from module representing PMF2. All data are presented as mean ± SEM. ** *p* < 0.01; ns, no significance, *p* ≥ 0.05.

Given that a tight junction is the main component of the intestinal barrier, a defective tight junction can lead to pathogen damage and runaway inflammation. We then explored the impact of breastfeeding and different formula feedings on the cell junctions of small intestinal organoids. First, we grouped all formula feeds into one group and all breast milk into another group to perform GSEA of cell junction-related pathways compared to the control group. The NES value of different pathways in different groups showed enrichment in cell junction-related pathways for both the formula feeding group and the breast milk feeding group, but overall, breast milk performed relatively better (Figure 4a). Furthermore, the GSEA pathway enrichment result predicted PMF2 performed the best, followed by PMF1 after comparing different formula feeds (Figure 4b).

We then focused on tight junctions, which are a primary part of the intestine barrier (Supplementary Figure S6a). We validated the mRNA expression of claudin-1 and ZO-1 proteins by RT-qPCR (Figure 4c, Supplementary Figure S4b). We found that, except for PMF2, compared to the control group, CLDN1 expression was not significantly affected by formula feeding, while TJP1 expression was significantly promoted by formula feeding. Similar to the discussion earlier about BM, significant heterogeneity was observed in the expression of ZO-1 and claudin-1 in BM groups. The hierarchical clustering heat map of GSEA core enrichment gene expression for each infant formulae in tight junctions (Figure 4d). Through the analysis, we identified three gene modules representing genes with relatively higher expression for a particular formula. We found that PMF2 had more genes with relatively higher expression. For each module’s genes, we predicted their protein interactions through STRING (Figure 4e). We identified hub genes from the interaction network and found metabolites with statistically high correlations (Supplementary Figure S6b–d). The expression of relevant key genes was validated by RT-qPCR (Supplementary Figure S6e–g), which showed the expression of infant formulae in the treatment group was significantly higher than the control group.

### 3.5. Extracellular Matrix Processes of Small Intestine Organoids Feeding by Different Infant Formulae and Breast Milk

**Figure 5:**
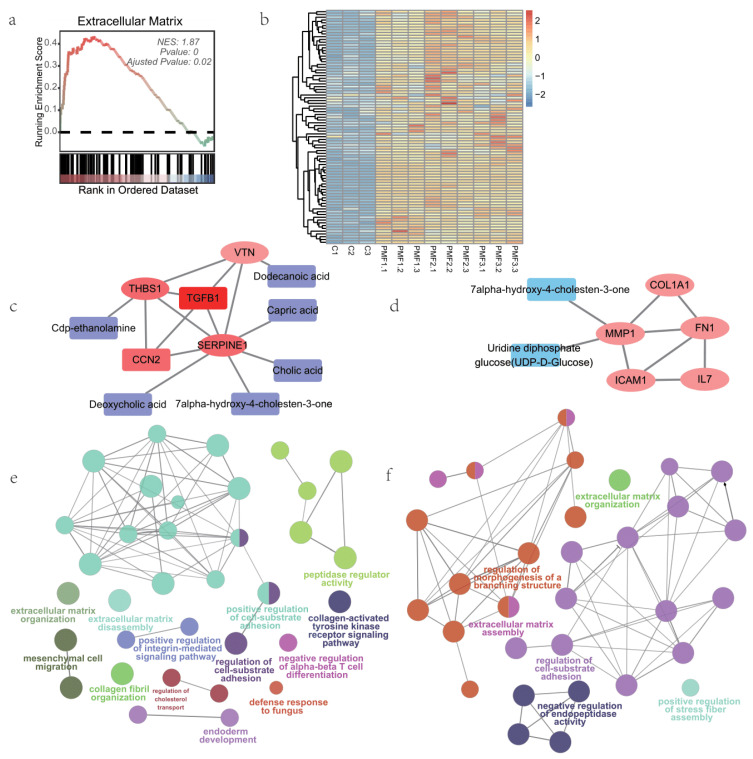
Profiles of extracellular events of breast milk and infant formulae. (**a**) GSEA result for GO (CC, cell compartment): Extracellular matrix obtained from identified shared DEG of breast milk and different infant formulae. (**b**) Heatmap of expression of core enrichment genes in Extracellular Matrix (ECM) generated from GSEA. (**c**) “Hub genes” and their highly correlated metabolites identified from network of ECM-related DEGs of BM. (**d**) “Hub genes” and their highly correlated metabolites identified from network of ECM-related DEGs of PMF. (**e**) Major GO terms enriched from ECM-related DEG of BM in the form of functionally grouped networks. (**f**) Major GO terms enriched from ECM-related DEG of PMF in the form of functionally grouped networks, similar terms were fused by clueGO.

We noticed that the GSEA of common DEGs between BM and PMF groups was significantly enriched in the Cellular Component (CC): Extracellular Matrix (Figure 5a), while there was no apparent difference between formula groups (Figure 5b). We then explored the differences between breast milk and formula in extracellular matrix processes. For breast milk and formula groups, we obtained their protein interaction networks for extracellular matrix-related genes through STRING. We identified key nodes, i.e., hub genes, in the network. Through Spearman correlation analysis, we obtained metabolites highly correlated with hub genes (BM group’s hub gene-metabolite network, Figure 5c; PMF group’s hub gene-metabolite network, Figure 5d).

By performing GO enrichment analysis on extracellular matrix-related DEGs for breast milk and formula groups, we identified functionally grouped networks with terms as nodes linked based on their kappa score level. Both formula feeds and breast milk are enriched in extracellular matrix organization and cell-substrate adhesion. However, breast milk is enriched in pathways related to stromal cell migration, collagen fiber organization, cholesterol transport, tyrosine kinase activation of collagen proteinase, and immune responses (fungal defense response, αβ T cell differentiation inhibition). On the other hand, formula feeds are enriched in branching structure morphogenesis, fibril assembly, and intrapeptide inhibition (Figure 5e,f).

## 4. Discussion

### 4.1. Application of Induced Pluripotent Stem Cell-Derived Organoid Models

Although animal models and cancer cell lines are currently the mainstream in human physiological research, in recent years, organoids have been increasingly used as in vitro models. They are self-organizing 3D structures consisting of stem cells and their more differentiated progeny [34]. For example, when fully mature, human intestinal organoids can differentiate into crypts and villi structures, carrying both stem cells and differentiated intestinal cells, including goblet cells, paneth cells, enterocytes, and enteroendocrine cells [59]. Moreover, organoids can also be rapidly cryopreserved and expanded [60]. Organoids have contributed to recent advances in modeling and understanding intestinal inflammation and infection, intestinal epithelial homeostasis, nutrient transport and sensing, intestine hormone secretion, and intestinal cancer [61,62,63,64,65].

Despite these advantages, the usage of organoids in our study also has its limitations. As in vitro models, organoids lack the intercommunication between different organ systems found in the human body and do not include other non-epithelial intestinal cell types or the microbiome. Additionally, the crypt and villi structures of organoids still show differences from those in vivo. Enteroids also lack physiological fluid flow and mechanical stimuli such as peristalsis. What is more, due to organoids’ large and complex tissue-like structures, organoids are technically more challenging and time-consuming compared to traditional in vitro cell culture models. To better mimic the intestinal epithelium and its microenvironment in vivo, our future work will focus on exploring and refining co-culture systems that integrate intestinal organoids with other tissue organoids. Additionally, recent published research by Yung, Claire, et al. used epithelial stem cell derived enteroids to study human milk’s nutrition component [66], as our study uses embryonic stem cell line H1 to generate enteroids. More research may be needed to compare small intestinal organoids from different sources in the future. Furthermore, our study did not focus on morphological changes, which are not significantly varied in the relatively short term before and after cultivated with formulas/milk digestion solution. However, the expression level of related genes can be detected, and we do have identified genes and pathways that support formulas/milk’s function in promoting organoids’ proliferation, differentiation, and organization. Further work may be needed to elucidate the physiology and morphological changes brought by formulas/milk’s nutrition.

Moreover, a hot topic in intestinal research is the study of the intestinal microbiome, and organoids derived from ESCs or pluripotent stem cells can also be used to simulate the interaction between intestinal epithelium and microbiome in vitro [67]. And the important mechanism through which breast milk or formula affects infant development is the intestinal microbiome [68]. Though we did not include microbiome in our organoid research in this study, we believed that future work would be conducted using enteroids to study interaction between infant formula/breast milk, microbiome, and nutrition effects.

### 4.2. Nutritional Absorption Effects Revealed by Transcriptomics and Metabolomics of Formula Feeding

In this study, we primarily investigated the overall nutrition effect of breastmilk (postpartum lactation period of 0–6 months) and infant formulas (stage-1) through pathway enrichment methods. Intriguingly, lipid-related pathways were enriched for all PMF groups but showed no significant difference while their related additive ingredients including inositol and L-carnitine have different amounts. We also observed that PMF groups showed better mineral absorption than BM groups, such as calcium. The additive component OPO (merely the same amounts added, see Table S1) may contribute [69]. Breast milk groups’ mineral absorption varied and was generally not as good as infant formula groups, which is consistent with previous studies that breast milk may not have enough zinc ion, ferric ion, and vitamin D [70,71,72], as the active form of vitamin D, 1,25(OH)2D, promotes calcium absorption in the small intestine [73]. However, PMF2 did not show significantly better calcium absorption than other infant formula groups as it also added another pro-calcium absorption ingredient, casein phosphopeptides.

Through shared DEG of formulas and breast milks, essential pathways of tissue development were enriched. Pathways such as the cell cycle and hippo signaling were engaged in tissue renewal and development. AMPK and mTOR were engaged in regulation of cell metabolism and development. Endocytosis is important in cellular metabolism and signal transduction [74]. The tight junction is the major component of the intestine barrier system, and it can be promoted by AMPK signaling [75]. TNF signaling and NOD-like receptor signaling are important parts of the innate immune system. What is more, enrichment of important metabolism was identified by untargeted metabolomics. Both formulas and breast milk enriched glycerophospholipid metabolism as well as ascorbate and aldarate metabolism. Glycerophospholipids are found in the highest amounts in the membranes of all cells and are a source of physiologically active compounds like eicosanoids, which engage in innate immunity [76]. Ascorbate and aldarate metabolism are a central pathway involving various conversions of glucose, including nucleotide synthesis and pentose interconversion. We then studied the more profound impact of breast milk and different infant formulae feeding on intestinal growth and development, cell connections, and the extracellular matrix.

Concerning growth and development, we identified the enrichment of the canonical Wnt pathway shared between breast milk and formula-fed groups. We also explored several gene-ontology biological processes such as cilium assembly, epithelial tube formation, and gland development. Therefore, we identified related genes including MUC2, ALPI, CHGA, STK3, WWTR1, and SMAD3, whose expressions were further validated using RT-qPCR. MUC2 is a marker of goblet cells in the small intestine. ALPI (intestinal alkaline phosphatase) serves as a marker for enterocytes, commonly located at the brush border of the intestinal mucosa. It induces the expression of proteins such as zonula-occludens, claudin, and occludin, maintaining the mechanical barrier of the intestinal mucosa. Simultaneously, it can also maintain the normal function of the intestinal mucosal barrier through the TLR4/NF-κB-mediated pathway [77,78,79,80]. CHGA (Chromogranin A) is a marker for enteroendocrine cells, and intestinal secretory cells play a role in various processes such as intestinal function, the secretion of intestinal hormones like insulin, and nutrient absorption [81,82]. STK3 and WWTR1, enriched from the GO-canonical Wnt pathway, are identified through a protein-protein interaction network, indicating their involvement in the regulation of growth and development. Through gene expression and pathway enrichment, our findings suggest that formula feeding, particularly certain infant formulae, not only can be comparable to but even surpasses breast milk in promoting overall growth and development, as significant variations between nutrition effects of breast milk groups were found in our study, which is consistent with the consensus that breast milk’s compositions could vary between donors depending on their individual factors, including maternal diet, health, genetics, environmental exposures, and age of the infant [83,84,85]. However, limitations of the organoids model might contribute to the results, as enteroids could be less differentiated than in the in vivo condition of a 0–6 month infant’s small intestine and more resemble the in vivo condition of preterm infants. The shortcomings of exclusively using breast milk for feeding preterm infants, such as deficiencies in certain nutrients or insufficient energy supply, have already been widely reported [86,87]. Future work is needed to comprehensively elucidate these results.

We specifically focused on the GO biological processes of cilium assembly, microvillus assembly and epithelial tube formation. Cilium serves as a crucial signaling center, regulating various pathways and facilitating interactions between the cell and its environment, contributing to maintaining tissue homeostasis, particularly in response to mechanical loading, extracellular subtrates, and releasing bioactive vesicles called ciliary ectosomes [88,89,90]. Microvilli expand the surface area for nutrient absorption in the small intestine, playing a crucial role in a range of small intestine functions [91,92]. Epithelial tube formation is a general term encompassing processes such as the maturation of the intestinal development and the morphogenesis of glandular structures in the intestine [93]. We identified differentially expressed genes associated with epithelial tube formation, along with metabolites that may reflect the regulation of gene transcription by metabolites or the regulation of metabolites by transcription [94]. SAMHD1 is highly correlated with cilium assembly while correlating with multiple metabolites. SAMHD1 is a deoxynucleoside triphosphate (dNTP) hydrolase that plays a crucial role in DNA double-strand break repair, genome stability, and the replication stress response in interferon-induced antiviral immunity (such as activation of the MRE11 nuclease to degrade the nascent DNA strand, preventing inflammation) [95,96]. RAP2A is highly correlated with microvillus assembly and a series of metabolites. It can perform as a molecular switch and mediate mechanoresponses of the Hippo pathway, while its overrepresentation induces cytoplasmic translocation of YAP and TAZ [97]. RAPGEF2 participates in development processes such as the nervous system, and it engages the RAPGEF2-RAP1A-ERK-cJUN axis, thus regulating the assembly of structures such as the adherence junction and proteins like β-catenin, E-cadherin, and ZO-1 [98,99].

For cell junctions, infant formula groups were generally not as good as breast milk groups, which is consistent with previous studies that breast milk feeding leads to less intestinal permeability [86,100]. We then focused on tight junctions. Tight junctions fulfill two major roles: (i) tight junctions prevent the mixing of membrane components; and (ii) tight junctions regulate the selective paracellular permeability. Disruption of tight junctions is regarded as one of the earliest hallmarks of epithelial injury, leading to the loss of cell polarity and tissue disorganization [101]. For the components of breast milk, human milk oligosaccharides (HMOs) have been shown to improve barrier function by modulating expression of tight junction proteins, thereby reducing permeability of the intestinal barrier [102] and contributing to our results. As low-cost alternatives to HMOs, oligofructose and oligo-galactose have also been demonstrated in the CACO-2 cell line to promote tight junctions [103]. This study employed organoid models and transcriptomics to investigate the supportive effects of different formula milks (containing oligofructose or oligo-galactose) and breast milk on tight junctions. We identified tight junction-related genes that were relatively highly expressed in different formula milks, explored their protein-protein interactions, obtained key genes in their interaction networks, and identified metabolites highly correlated with them. For PMF1, KRAS is involved in various processes regulating cell proliferation and growth, such as the MAPK pathway, mTOR pathway, and PI3K-AKT signaling pathway [104]. FN1 encodes fibronectin 1, a glycoprotein that can activate the Wnt/β-catenin pathway [105]. For PMF2, ITGA2 is necessary for cell survival, reproduction, and migration [106], and its correlated metabolites directly or indirectly participate in lipid metabolisms (dodecanoic acid, capric acid, deoxycholic acid, and cholic acid). CAV1 is a membrane scaffolding protein associated with caveolae biogenesis, cholesterol transport, endocytosis, cell signaling, and other cellular processes [107]. Acetylcarnitine and gamma-muricholic acid, which are highly correlated with CAV1, are metabolites directly or indirectly involved in lipid metabolism. Dietary betaine, as a methyl donor, can support cellular methionine homeostasis and has broad biological benefits [108]. For PMF3, LIN7C is involved in widespread epithelial cell polarity maturation [109], and GJA1 is one of the most ubiquitously expressed junction proteins and participates in a wide range of biological processes [110].

For the extracellular matrix, previous research has indicated that oligosaccharides such as low-dose galacto-oligosaccharides upregulate the expression of genes and proteins related to human goblet cells, thereby regulating the secretion function of goblet cells and enhancing intestinal barrier function [111]. Components of the extracellular matrix, such as hyaluronic acid and chondroitin sulfate, play crucial roles in immune system regulation and defense [112,113,114,115]. The main extracellular matrix events observed here are related to collagen and peptidase. Collagen proteins form a three-dimensional extracellular scaffold, providing binding sites for many ECM glycoproteins and soluble growth factors. They play a crucial role in cell-ECM interactions, potentially promoting cell survival [116,117,118]. However, further research is needed to understand more about the additional roles of collagen proteins and the distribution of collagen types in this context. The action of peptidases may be associated with the remodeling of the extracellular matrix and tissue structure, participating in the proliferation, growth, development, and migration of tissue cells.

In this study, the nutritional absorption effects of different formula milks and breast milk were compared using transcriptomics and non-targeted metabolomics approaches with an induced embryonic stem cell-derived small intestinal organoid model. Infant formulae demonstrated comparable effects to breast milk in overall nutrition absorption, growth, and development. However, differences and disadvantages persisted in terms of cell connectivity and extracellular matrix when compared to breast milk. In these three aspects, we identified the transcriptomic pathway annotations, metabolic pathways, and interaction networks of different sample groups, providing crucial information for the study of nutritional effects. In our study, breast milk’s compositions were inferred from previous research, while infant formulas’ compositions were obtained from publicly available ingredient tables and information attached with these products. Future works will focus on precisely quantifying the components and their structures of infant formula and breast milk, as well as identifying the factors that influence macronutrient effects on the infant small intestine. Nevertheless, further and more detailed research is required to elucidate the short-term or long-term effects of breast milk and formula milk, either overall or with respect to specific components, on the infantile small intestine.

## 5. Conclusions

In this study, we compared the nutritional absorption effects of different infant formulas and breast milk in an embryonic stem cell-induced small intestine organoid model, using transcriptomics and untargeted metabolomics methods. Current stage-1 infant formulas can match the overall growth and development effects of breast milk (postpartum lactation period of 0–6 months). Using pathway enrichment score and expression key genes (e.g., SMAD3, WWTR1) as references, we demonstrated that breast milk’s pro-development effect varies between donors significantly, while infant formula groups performed better compared to some breast milk groups. However, infant formulas still generally show differences and deficiencies in terms of cell junctions and extracellular matrix compared to breast milk. In these three aspects, we identified transcriptomic pathway annotations, metabolomic metabolic pathways, and interaction networks for the different sample groups. Though the three infant formulas have similar main ingredients, many unique characteristics were identified. For instance, PMF1 was linked to peroxisomal membrane transport, PMF2 was characterized by ionotropic glutamate receptor activity, and PMF3 was enriched in response to vitamin D and T-cell-mediated immunity. We also predicted genes highly expressed in certain formula groups that may operate in each group’s different nutrition effect, such as KRAS1 for PMF1, ITGA2 for PMF2, and LIN7C for PMF3. These results may help further nutrition effect studies.

## Data Availability

The original contributions presented in the study are included in the article, further inquiries can be directed to the corresponding author.

## Supplementary Materials

The following supporting information can be downloaded at: https://www.mdpi.com/article/10.3390/nu16172951/s1, Figure S1: Normalization and identification of differentially expressed genes following bulk RNA-seq, Figure S2: OPLS-DA analysis for inter-infant formulae comparison, Figure S3: Correlation of infant formulae pair-wise comparison and differences of breast milk metabolomics, Figure S4: Heatmap of expression of core enrichment genes, Figure S5: Network regulating growth and development predicted by IPA for each group, Figure S6: Exploration of tight junction and its regulation for each group; Table S1: Ingredient table of infant formulas, Table S2: gradient of fluid phae during elution, Table S3: primer sequences used in RT-qPCR, Table S4: Enriched Pathways from Comparisons between groups.

## Abbreviations

ESC	embryo stem cell
FA%	% of fatty acids
DHA	Docosahexaenoic acid
ARA	arachidonic acid
CACO-2	a widely used human colorectal adenocarcinoma cell line, simulating intestine epithelial cells
BM	breast milk
PMF1, PMF2, PMF3 (p1, p2, p3)	experimental groups in our study, representing infant formulas groups
BM1~5 (b1~5)	experimental groups in our study, representing breast milk groups
DEG	differential expressed genes
GSVA	Gene Set Variation Analysis
GSEA	Gene Set Enrichment analysis
KEGG	kyoto encyclopedia of genes and genomes
GO	gene ontology
IPA	Ingenuity Pathway Analysis, a software application for analyzing omics data
STRING	a database of functional protein association networks
OPO	1,3-Dioleoyl-2-palmitoyl glycerol
ECM	extracellular matrix
HMO	Human Milk Oligosaccharides
SEM	standard error of the mean
NES	normalized enrichment score
