# Supplementary material for: Multi-Omics Profiles of Small Intestine Organoids in Reaction to Breast Milk and Different Infant Formula Preparations
Source: Nutrients. 2024 Sep 2;16(17):2951. doi: 10.3390/nu16172951 (PMC11397455; doi:10.3390/nu16172951)
Supplement: Supplementary file 1 [file nutrients-16-02951-s001.zip › nutrients-3134820-supplementary.pdf]

Supplementary Material

Multi-omics profiles of small intestine organoids in reaction to breast milk and different infant formula preparations

Xianli Wang[a], Shangzhi Yang[b], Chengdong Zheng[c], Chenxuan Huang[b], Haiyang Yao[b], Zimo Guo[b], Yilun Wu[b], Zening Wang[d], Zhenyang Wu[b], Ruihong Ge[a], Wei Cheng[a], Yuanyuan Yan[a], Shilong Jiang[c], Jianguo Sun[c], Jingquan Li[a], Qinggang Xie[c], Xiaoguang Li[e], Hui Wang[e].

Co-first Authors:

Xianli Wang[a], Shangzhi Yang[b], Chengdong Zheng[c]

Corresponding Authors:

Qinggang Xie[c], E-mail: xieqinggang@feihe.com

Hui Wang[e], E-mail: huiwang@shsmu.edu.cn

a. School of Public Health, Shanghai Jiao Tong University School of Medicine, Shanghai 200025, China.

b. School of Medicine, Shanghai Jiao Tong University, Shanghai 200025, China

c. Heilongjiang Firmus Dairy Co., Ltd., C-16, 10A Jiuxianqiao Rd., Chaoyang, Beijing 100015, China

d. Institutes of Biomedical Sciences, Fudan University, 131 Dongan Road, Shanghai, 200032, China

e. State Key Laboratory of Systems Medicine for Cancer, Center for Single-Cell Omics, School of Public Health, Shanghai Jiao Tong University School of Medicine, Shanghai 200025, China.

Figure captions

Supplementary Figure 1 Normalization and identification of differential expressed genes following bulk RNA-seq. a Violin plot of quantified gene expression after normalization to remove library size effect. **b** Heatmap of normalized gene expression of each sample, all values are row-wisely scaled as z-score. **c** Volcano plots visualize the differential expressed genes. Genes satisfying p.adjust < 0.05 and foldchange > 2 are colored in red, while genes satisfying p.adjust < 0.05 and foldchange < 0.5 are colored in blue. Other genes are colored in grey, which means they are not significantly different.

Supplementary Figure 2 OPLS-DA analysis for inter-infant-formulae comparison. **a-c** Pair wise OPLS-DA score plots of the LC-MS data in positive and negative ionization mode: (**a**) PMF1 vs PMF2, (**b**) PMF1 vs PMF3, (**c**) PMF2 vs PMF3. R2X and R2Y respectively represent the explanatory power of the model for the X and Y matrices, while Q2 indicates the predictive ability of the model. These three indicators collectively demonstrate the effectiveness of the model. **d** S-plot generated from OPLS-DA of inter-infant-formulae comparison. S-plot visualize the modelled covariance (X-

axis) and correlation (Y-axis). The variables showing the highest p and p (corr) absolute value are considered the most relevant variable (metabolites) for classification of samples.

Supplementary Figure 3 Correlation of infant formulae pair-wise comparison and differences of breast milk metabolomics. a-c Heatmap of spearman correlation analysis of differential metabolites from three pairwise comparison of (**a**) PMF1 vs PMF2, (**b**) PMF1 vs PMF3, (**c**) PMF2 vs PMF3. **d** Heatmap of normalized metabolite level of different breast milk group.

Supplementary Figure 4 Heatmap of expression of core enrichment genes. **a** Core enrichment genes in cilium assembly. **b** RT-qPCR quantifies "key genes" expression in each group of breast milk ($2^{-\Delta\Delta Ct}$ method). Heatmap was drawn by row-wise z-score scaled log($2^{-\Delta\Delta Ct}$) value. Grey indicates absent values. **c** Core enrichment genes in epithelial tube formation.

Supplementary Figure 5 Network regulating growth and development predicted by IPA for each group: (**a**) BM, (**b**) PMF1, (**c**) PMF2, (**d**) PMF3. Red node indicates increased measurement while green node indicates decreased measurement. Orange node indicates predicted activation while blue node indicates predicted inhibition. Glow around node indicates activity when opposite of measurement. Orange edge leads to activation, blue edge leads to inhibition, and yellow edge means findings inconsistent with state of downstream molecule.

Supplementary Figure 6 Exploration of tight junction and its regulation for each group. a KEGG map visualizing the gene expression of breast milk and different infant formulae compared to control group. Red means Fold Change > 2. Pink means 1 < Fold Change < 2. Cyan means 1/2 < Fold Change < 1. Blue means Fold Change < 1/2. All input genes were DEG satisfying p.adj ust< 0.05. **b-d** 'Hub genes' identified from protein-protein interaction network, and metabolites highly correlated (spearman correlation > 0.8) to these "hub genes" were visualized respectively for PMF1 (**b**), PMF2 (**c**), and PMF3 (**d**) . **e-g** RT-qPCR quantifies (by $2^{-\Delta\Delta Ct}$ method) expressions of "key genes" in the correlation network respectively for PMF1 (**e**), PMF2 (**f**), and PMF3 (**g**). In the bar chart, mean values with SEM were illustrated.

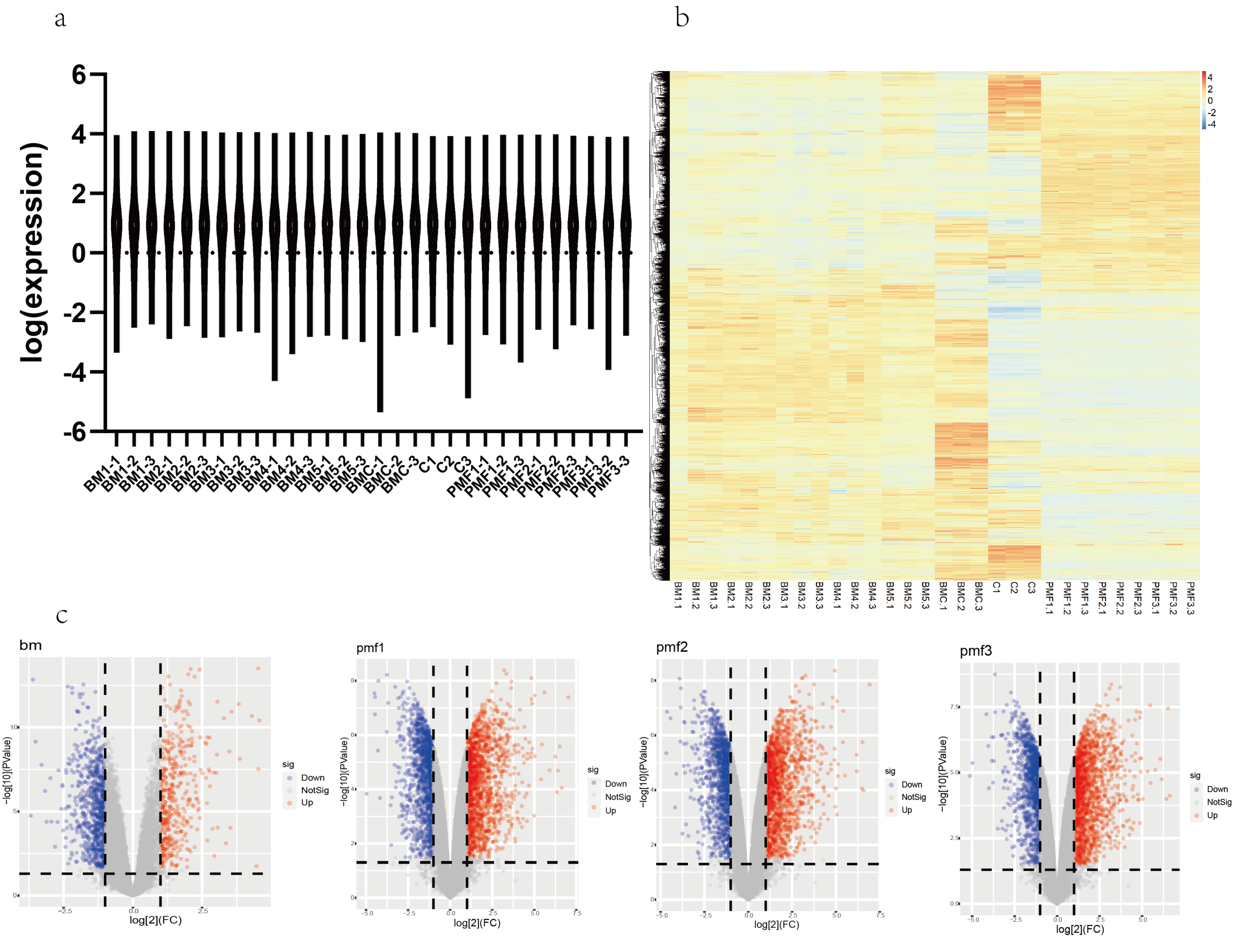
a
log(expression)
BM1-1 BM1-2 BM1-3 BM2-1 BM2-2 BM2-3 BM3-1 BM3-2 BM3-3 BM4-1 BM4-2 BM4-3 BM5-1 BM5-2 BM5-3 BMC-1 BMC-2 BMC-3 C1 C2 C3 PMF1-1 PMF1-2 PMF1-3 PMF2-1 PMF2-2 PMF2-3 PMF3-1 PMF3-2 PMF3-3
b
BM1.1 BM1.2 BM1.3 BM2.1 BM2.2 BM2.3 BM3.1 BM3.2 BM3.3 BM4.1 BM4.2 BM4.3 BM5.1 BM5.2 BM5.3 BMC.1 BMC.2 BMC.3 C1 C2 C3 PMF1.1 PMF1.2 PMF1.3 PMF2.1 PMF2.2 PMF2.3 PMF3.1 PMF3.2 PMF3.3
c
bm
pmf1
pmf2
pmf3
−log[10](PValue)
log[2](FC)
sig
Down
NotSig
Up

Figure S1

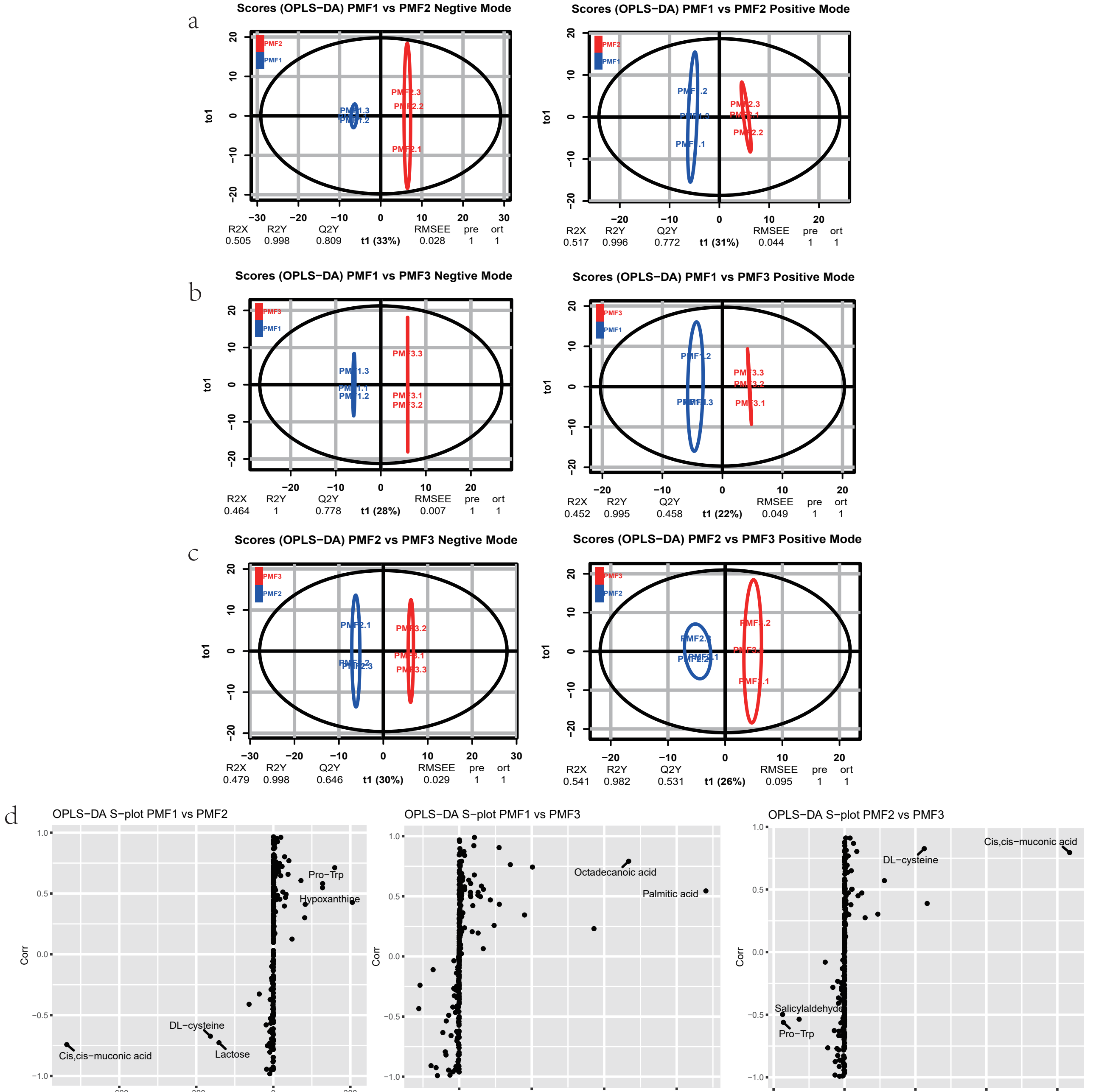

a
Scores (OPLS−DA) PMF1 vs PMF2 Negtive Mode
PMF2
PMF1
t1 (33%)
R2X 0.505
R2Y 0.998
Q2Y 0.809
RMSEE 0.028
pre 1
ort 1
Scores (OPLS−DA) PMF1 vs PMF2 Positive Mode
t1 (31%)
R2X 0.517
R2Y 0.996
Q2Y 0.772
RMSEE 0.044
pre 1
ort 1
b
Scores (OPLS−DA) PMF1 vs PMF3 Negtive Mode
PMF3
PMF1
t1 (28%)
R2X 0.464
R2Y 1
Q2Y 0.778
RMSEE 0.007
pre 1
ort 1
Scores (OPLS−DA) PMF1 vs PMF3 Positive Mode
t1 (22%)
R2X 0.452
R2Y 0.995
Q2Y 0.458
RMSEE 0.049
pre 1
ort 1
c
Scores (OPLS−DA) PMF2 vs PMF3 Negtive Mode
PMF3
PMF2
t1 (30%)
R2X 0.479
R2Y 0.998
Q2Y 0.646
RMSEE 0.029
pre 1
ort 1
Scores (OPLS−DA) PMF2 vs PMF3 Positive Mode
t1 (26%)
R2X 0.541
R2Y 0.982
Q2Y 0.531
RMSEE 0.095
pre 1
ort 1
to1
d
OPLS−DA S−plot PMF1 vs PMF2
Pro−Trp
Hypoxanthine
DL−cysteine
Lactose
Cis,cis−muconic acid
OPLS−DA S−plot PMF1 vs PMF3
Octadecanoic acid
Palmitic acid
OPLS−DA S−plot PMF2 vs PMF3
Cis,cis−muconic acid
DL−cysteine
Salicylaldehyde
Pro−Trp
Corr
Cov

Figure S2

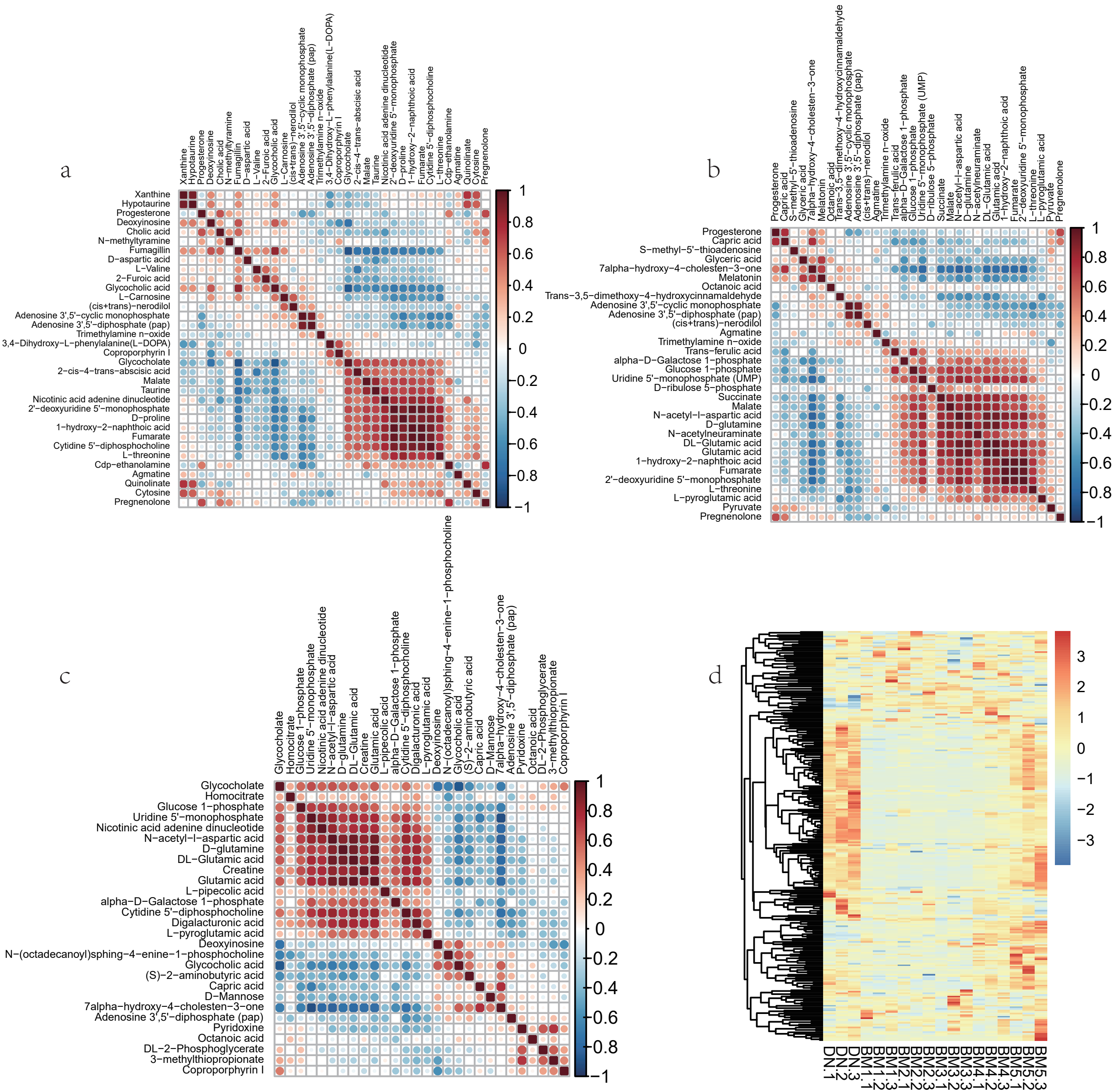
a
Xanthine
Hypotaurine
Progesterone
Deoxyinosine
Cholic acid
N−methyltyramine
Fumagillin
D−aspartic acid
L−Valine
2−Furoic acid
Glycocholic acid
L−Carnosine
(cis+trans)−nerodilol
Adenosine 3',5'−cyclic monophosphate
Adenosine 3',5'−diphosphate (pap)
Trimethylamine n−oxide
3,4−Dihydroxy−L−phenylalanine(L−DOPA)
Coproporphyrin I
Glycocholate
2−cis−4−trans−abscisic acid
Malate
Taurine
Nicotinic acid adenine dinucleotide
2'−deoxyuridine 5'−monophosphate
D−proline
1−hydroxy−2−naphthoic acid
Fumarate
Cytidine 5'−diphosphocholine
L−threonine
Cdp−ethanolamine
Agmatine
Quinolinate
Cytosine
Pregnenolone
1
0.8
0.6
0.4
0.2
0
−0.2
−0.4
−0.6
−0.8
−1
b
Progesterone
Capric acid
S−methyl−5'−thioadenosine
Glyceric acid
7alpha−hydroxy−4−cholesten−3−one
Melatonin
Octanoic acid
Trans−3,5−dimethoxy−4−hydroxycinnamaldehyde
Adenosine 3',5'−cyclic monophosphate
Adenosine 3',5'−diphosphate (pap)
(cis+trans)−nerodilol
Agmatine
Trimethylamine n−oxide
Trans−ferulic acid
alpha−D−Galactose 1−phosphate
Glucose 1−phosphate
Uridine 5'−monophosphate (UMP)
D−ribulose 5−phosphate
Succinate
Malate
N−acetyl−l−aspartic acid
D−glutamine
N−acetylneuraminate
DL−Glutamic acid
Glutamic acid
1−hydroxy−2−naphthoic acid
Fumarate
2'−deoxyuridine 5'−monophosphate
L−threonine
L−pyroglutamic acid
Pyruvate
Pregnenolone
1
0.8
0.6
0.4
0.2
0
−0.2
−0.4
−0.6
−0.8
−1
c
Glycocholate
Homocitrate
Glucose 1−phosphate
Uridine 5'−monophosphate
Nicotinic acid adenine dinucleotide
N−acetyl−l−aspartic acid
D−glutamine
DL−Glutamic acid
Creatine
Glutamic acid
L−pipecolic acid
alpha−D−Galactose 1−phosphate
Cytidine 5'−diphosphocholine
Digalacturonic acid
L−pyroglutamic acid
Deoxyinosine
N−(octadecanoyl)sphing−4−enine−1−phosphocholine
Glycocholic acid
(S)−2−aminobutyric acid
Capric acid
D−Mannose
7alpha−hydroxy−4−cholesten−3−one
Adenosine 3',5'−diphosphate (pap)
Pyridoxine
Octanoic acid
DL−2−Phosphoglycerate
3−methylthiopropionate
Coproporphyrin I
1
0.8
0.6
0.4
0.2
0
−0.2
−0.4
−0.6
−0.8
−1
d
3
2
1
0
−1
−2
−3
DN.1
DN.2
DN.3
BM1.1
BM1.2
BM1.3
BM2.1
BM2.2
BM2.3
BM3.1
BM3.2
BM3.3
BM4.1
BM4.2
BM4.3
BM5.1
BM5.2
BM5.3

Figure S3

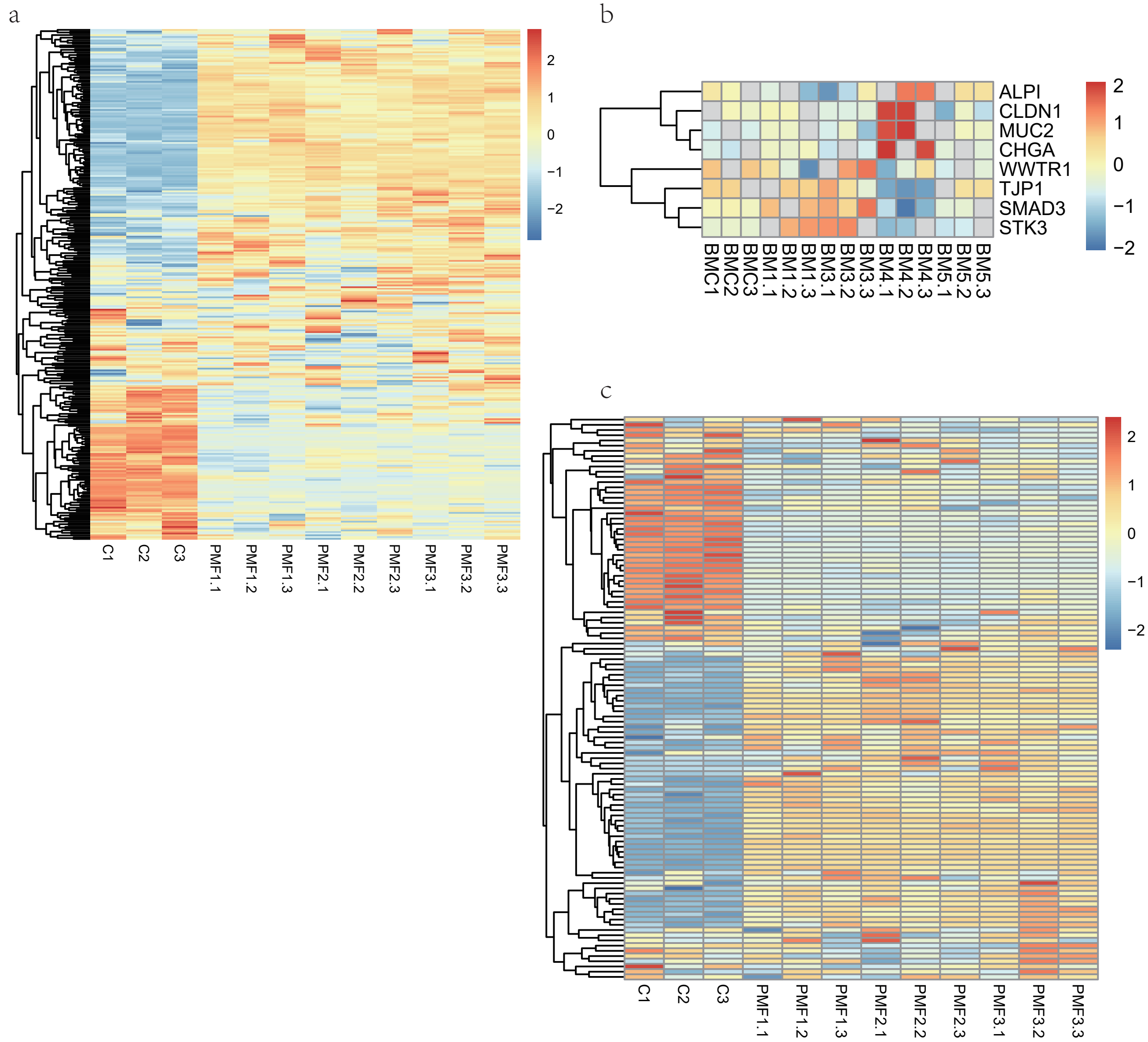
a
b
c
ALPI
CLDN1
MUC2
CHGA
WWTR1
TJP1
SMAD3
STK3
BMC1
BMC2
BMC3
BM1.1
BM1.2
BM1.3
BM3.1
BM3.2
BM3.3
BM4.1
BM4.2
BM4.3
BM5.1
BM5.2
BM5.3
C1
C2
C3
PMF1.1
PMF1.2
PMF1.3
PMF2.1
PMF2.2
PMF2.3
PMF3.1
PMF3.2
PMF3.3

Figure S4

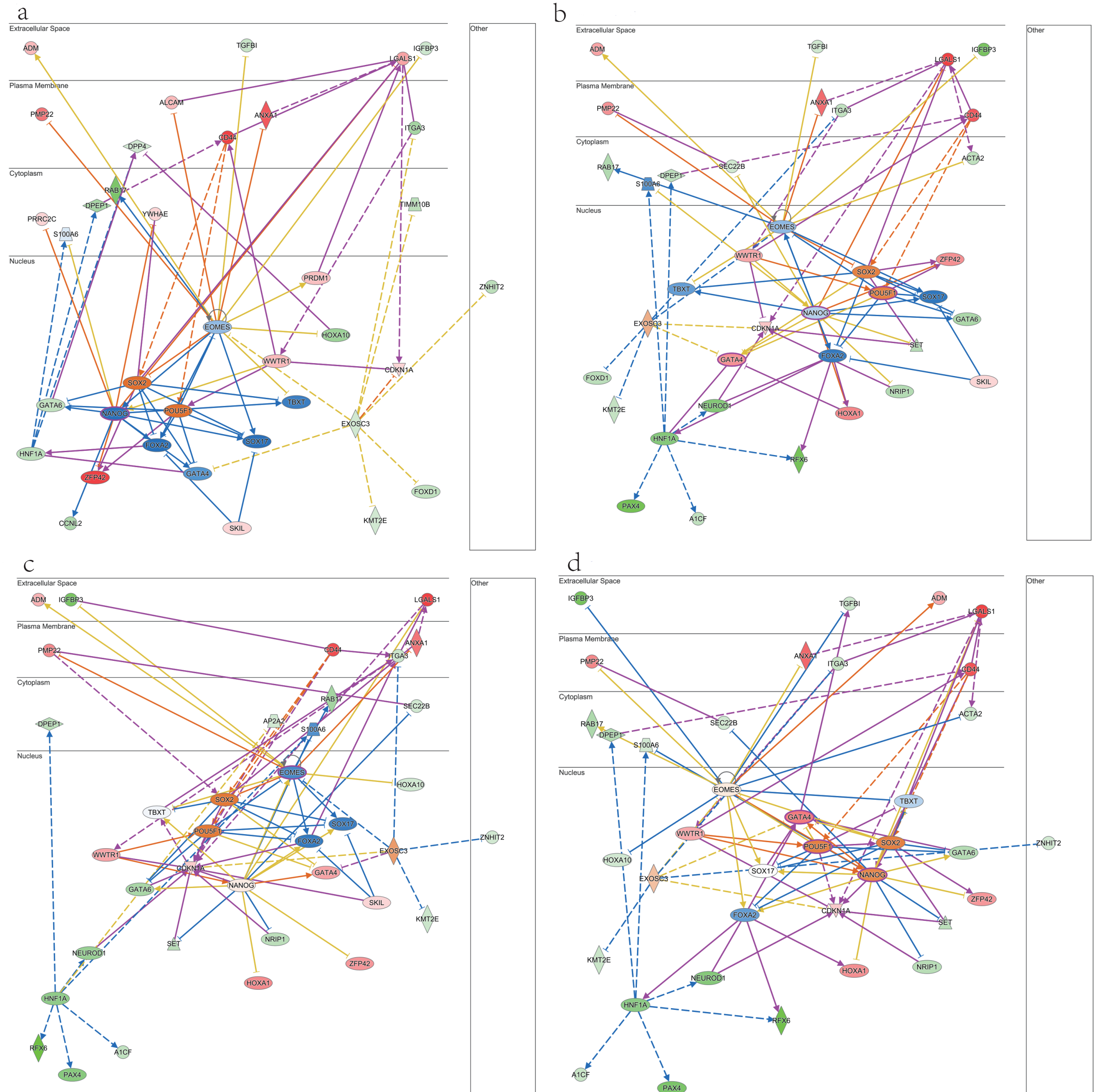
a
b
c
d
Extracellular Space
Plasma Membrane
Cytoplasm
Nucleus
Other
ADM
TGFBI
IGFBP3
LGALS1
PMP22
ALCAM
ANXA1
CD44
ITGA3
DPP4
RAB17
DPEP1
PRRC2C
YWHAE
TIMM10B
S100A6
PRDM1
ZNHIT2
EOMES
HOXA10
WWTR1
CDKN1A
SOX2
TBXT
GATA6
NANOG
POU5F1
EXOSC3
SOX17
FOXA2
HNF1A
GATA4
ZFP42
FOXD1
KMT2E
CCNL2
SKIL
SEC22B
ACTA2
NRIP1
SET
NEUROD1
HOXA1
RFX6
PAX4
A1CF
AP2A2

Figure S5

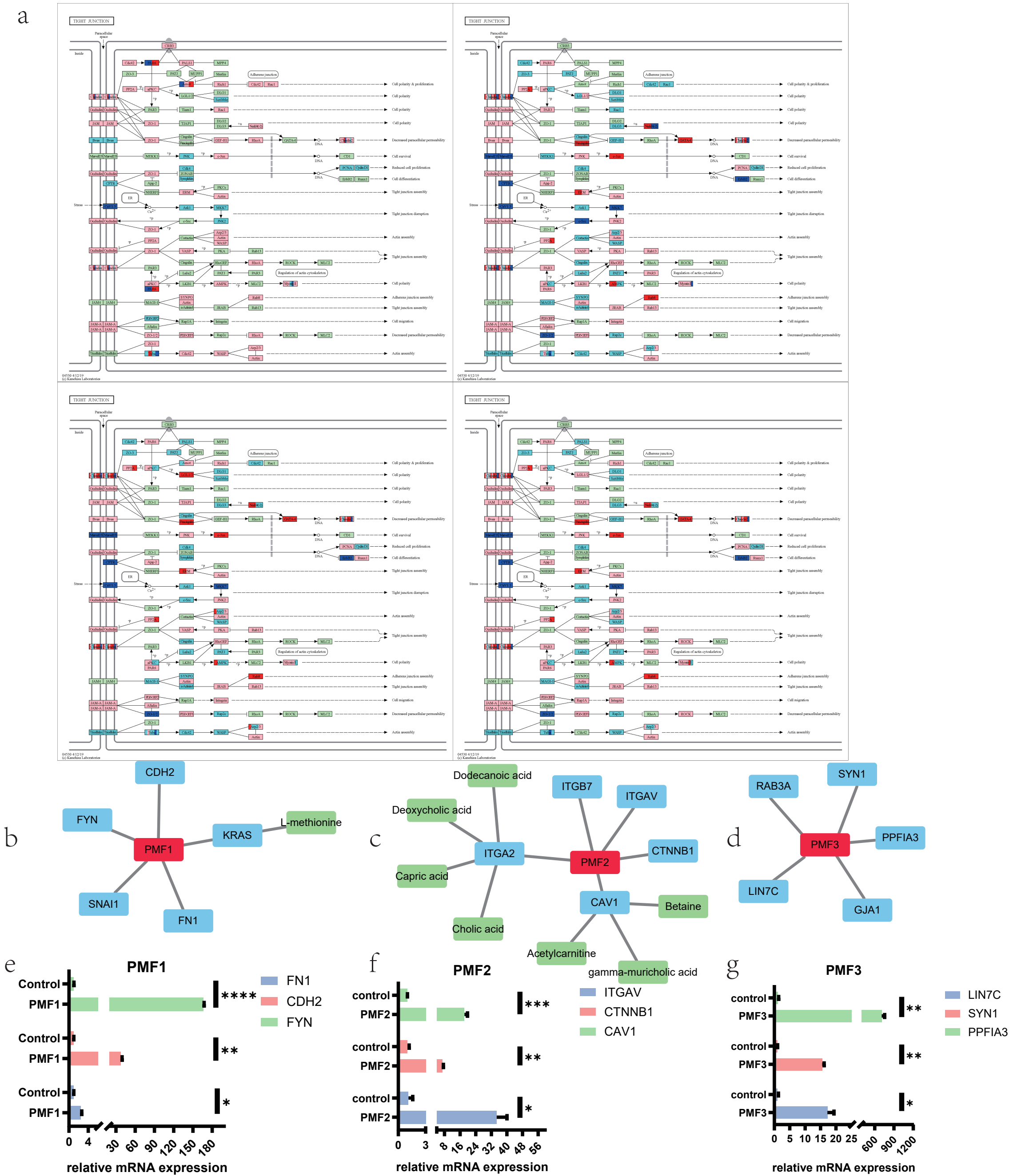
a
TIGHT JUNCTION
b
CDH2
FYN
KRAS
L-methionine
PMF1
SNAI1
FN1
c
Dodecanoic acid
Deoxycholic acid
ITGB7
ITGAV
ITGA2
PMF2
CTNNB1
Capric acid
CAV1
Betaine
Cholic acid
Acetylcarnitine
gamma-muricholic acid
d
RAB3A
SYN1
PMF3
PPFIA3
LIN7C
GJA1
e
PMF1
Control
PMF1
FN1
CDH2
FYN
****
**
*
0
4
30
60
90
120
150
180
relative mRNA expression
f
PMF2
control
PMF2
ITGAV
CTNNB1
CAV1
***
**
*
0
3
8
16
24
32
40
48
56
relative mRNA expression
g
PMF3
control
PMF3
LIN7C
SYN1
PPFIA3
**
**
*
0
5
10
15
20
25
600
900
1200
relative mRNA expression

Figure S6

Table S1: Ingredient table of infant formulas

component	measurement (per 100g)	PMF1	PMF2	PMF3
linoleic acid	mg	3800	2600	4100
α-linolenic acid	mg	423	260	410
vitaminA	μg RE	500	450	500
vitaminD	μg	7	12	7.5
vitaminE	mg α-TE	6	4.9	6
vitaminK1	μg	56	45	60
vitaminB1	μg	623	650	650
vitaminB2	μg	600	723	500
vitaminB6	μg	450	290	520
vitaminB12	μg	1.5	1.3	1.5
nicotinic acid	μg	4100	3600	4100
folic acid	μg	72	50	67
pantothenic acid	μg	3000	4300	3050
vitaminC	μg	72000	49000	70000
biotin	μg	16	12	16
Na	mg	131	150	131
K	mg	400	510	360
Cu	mg	0.36	0.315	0.35
Mg	mg	31	42	32
Fe	mg	4.5	6.8	5
Zn	mg	4	4	4.5
Mn	mg	0.043	0.037	0.04
Ca	mg	360	490	350
P	mg	210	330	200
I	mg	0.072	0.04	0.07
Cl	mg	297	310	280
Se	mg	0.015	0.012	0.015
choline	mg	65	200	215
inositol	mg	40	40	0
taurine	mg	38	38	0
L-carnitine	mg	9	11	0
DHA/(% total FA)		0.17	0.17	0.18
ARA/(% total FA)		0.31	0.35	0.36
Fructo-oligosaccharide	mg	1700	0	1500
OPO	mg	3800	4000	4000
lutein	mg	66	210	0
nucleotide	mg	30	30	0
Casein phosphopeptides	mg	0	40	0
Lactoferrin	mg	0	0	45
Galacto-oligosaccharides	mg	0	3000	0

Bifidobacterium animalis subsp. *lactis*, BB-12® (BB-12) was added in **PMF1**.

The ingredients of **PMF1,2** also include five maternal nucleotides (disodium 5'-cytidine monophosphate, disodium 5'-uridine monophosphate, 5'-adenosine monophosphate, disodium 5'-guanosine monophosphate, disodium 5'-inosine monophosphate).

Hydrolyzed whey protein powder was used in **PMF2**.

PMF3 has organic certification.

Table S2: gradient of fluid phase during elution

Time (min)	A (%)<br>water with 25 mM ammonium acetate and 25 mM ammonium hydroxide (%)	B(%)<br>acetonitrile (%)
0	5	95
0.5	5	95
7	35	65
8	60	40
9	60	40
9.1	5	95
12	5	95

Table S3: primer sequences used in RT-qPCR

Name	Sequence (5' to 3')
LYZ-F	CTTGTCCTCCTTTCTGTTACGG
LYZ-R	CCCCTGTAGCCATCCATTCC
MUC2-F	GAGGGCAGAACCCGAAACC
MUC2-R	GGCGAAGTTGTAGTCGCAGAG
ALPI-F	TGAGGGTGTGGCTTACCAG
ALPI-R	GATGGACGTGTAGGCTTTGCT
CHGA-F	TAAAGGGGATACCGAGGTGATG
CHGA-R	TCGGAGTGTCTCAAAACATTCC
CLDN1-F	CCTCCTGGGAGTGATAGCAAT
CLDN1-R	GGCAACTAAAATAGCCAGACCT
TJP1-F	CAACATACAGTGACGCTTCACA
TJP1-R	CACTATTGACGTTTCCCCACTC
TJP2-F	ATGGAAGAGCTGATATGGGAACA
TJP2-R	TGCTGAACTGCAAACGAATGAA
CLDN4-F	TGGGGCTACAGGTAATGGG
CLDN4-R	GGTCTGCGAGGTGACAATGTT
CDH2-F	TCAGGCGTCTGTAGAGGCTT
CDH2-R	ATGCACATCCTTCGATAAGACTG
FN1-F	CGGTGGCTGTCAGTCAAAG
FN1-R	AAACCTCGGCTTCCTCCATAA
SNAI1-F	TCGGAAGCCTAACTACAGCGA
SNAI1-R	AGATGAGCATTGGCAGCGAG
FYN-F	ATGGGCTGTGTGCAATGTAAG
FYN-R	GAAGCTGGGGTAGTGCTGAG
WWTR1-F	GATCCTGCCGGAGTCTTTCTT
WWTR1-R	CACGTCGTAGGACTGCTGG
STK3-F	CTTTGGTCCGATGATTTCACCG
STK3-R	GGATGCTGTAAAAGTTGTGTTGC
LIN7C-F	GAAGTGAGAGCGAACGCTACT
LIN7C-R	TCCACCTGGAATTATTCGGGAT
ITGAV-F	ATCTGTGAGGTCGAAACAGGA
ITGAV-R	TGGAGCATACTCAACAGTCTTTG
PPFIA3-F	ACAGGACGGGTTGGCTACA
PPFIA3-R	AAGTTCAGCTCCTTCGTCAGA
SYN1-F	AGTTCTTCGGAATGGGGTGAA
SYN1-R	CAAACTGCGGTAGTCTCCGTT

Table S4: Enriched Pathways from Comparison between groups

Group	Pathway	Total	Expected	Hits	Raw p	Holm p	Metabolites	LogFC	p
PMF1 vs PMF2	Taurine and Hypotaurine Metabolism	12	0.323	2	0.0391	1			
							hypotaurine	-0.30275	0.027888
							taurine	0.442957	0.008986
PMF2 vs PMF3	Lactose Synthesis	19	0.417	3	0.0071	0.695			
							Uridine 5'-monophosphate	0.310555445	0.046902243
							alpha-D-Galactose	0.911340799	0.000662839
							Glucose 1-phosphate	0.450403687	0.013480048
	Galactose Metabolism	38	0.834	3	0.0471	1			
							D-Mannose	-2.076851634	0.00184388
							alpha-D-Galactose 1-phosphate	0.911340799	0.000662839
							Glucose 1-phosphate	0.450403687	0.013480048
PMF1 vs PMF3	Warburg Effect	57	1.31	5	0.00763	0.747			
	Malate-Aspartate Shuttle	10	0.23	2	0.0203	1			
							Glutamic acid	0.342636311	0.026011108
							Malate	0.665481591	0.004061283
	Glucose-Alanine Cycle	13	0.298	2	0.0337	1			
							Glutamic acid	0.342636311	0.026011108
							Pyruvate	-1.166757554	0.026956254
	Citric Acid Cycle	32	0.735	3	0.0339	1			
							Pyruvate	-1.166757554	0.026956254

							Malate	0.665481591	0.004061283
							Succinate	0.347773362	0.03236625
	Gluconeogenesis	33	0.757	3	0.0368	1			
							Pyruvate	-1.166757554	0.026956254
							Malate	0.665481591	0.004061283
							Glucose 1-phosphate	0.450403687	0.013480048
	Glycine and Serine Metabolism	59	1.35	4	0.0414	1			
							Glyceric acid	-0.572844909	0.021215054
							Glutamic acid	0.342636311	0.026011108
							L-threonine	-0.402340237	0.048211226
							Pyruvate	-1.166757554	0.026956254
PMF1 vs BM	Purine Metabolism	73	0.437	3	0.00633	0.621			
							Glycine	-0.43897	0.001484
							Inosine	0.468097	0.018542
							Xanthosine	0.473431	0.027886
PMF2 vs BM	Purine Metabolism	73	0.51	3	0.0105	1			
							Glycine	-0.34619	0.015859
							Inosine	0.412384	0.032949
							Xanthosine	0.489477	0.025981
PMF3 vs BM	Glycine and Serine Metabolism	59	0.589	4	0.00175	0.171			
							Betaine	0.234328	0.041174
							Glycine	-0.38063	0.006222
							Glyceric acid	0.662103	0.005779
							Glutamic acid	-0.39145	0.014505
	Purine Metabolism	73	0.739	4	0.0039	0.378			

						Glycine	-0.38063	0.006222
						Glutamic acid	-0.39145	0.014505
						Inosine	0.412337	0.029163
						Xanthosine	0.443257	0.036041
Alanine Metabolism	17	0.17	2	0.0113	1			
						Glycine	-0.38063	0.006222
						Glutamic acid	-0.39145	0.014505
Lactose Synthesis	19	0.19	2	0.014	1			
						Galactose 1-phosphate	-0.61984	0.019582
						Glucose 1-phosphate	1.0981	0.026156
Nucleotide Sugars Metabolism	20	0.2	2	0.0155	1			
						Galactose 1-phosphate	-0.61984	0.019582
						Glucose 1-phosphate	1.0981	0.026156
Glutathione Metabolism	20	0.2	2	0.0155	1			
						Glycine	-0.38063	0.006222
						Glutamic acid	-0.39145	0.014505

	Ammonia Recycling	31	0.309	2	0.0357	1			
							Glutamic acid	-0.39145	0.014505
							Glycine	-0.38063	0.006222

*Metabolites enrichment analysis conducted by Metabolanalyst (**https://www.metaboanalyst.ca/**) using Enrichment Analysis module and SMPDB database.*
